# Investigating the relationship between Toll-like receptor activity, low-grade inflammation, cognitive deficits, and antipsychotic drug dose in schizophrenia patients: a moderation analysis

**DOI:** 10.1017/S0033291726103596

**Published:** 2026-03-03

**Authors:** Saahithh Redddi Patlola, Brian Hallahan, Ross McManus, Marcus Kenyon, Colm McDonald, Derek Morris, John Kelly, Gary Donohoe, Declan P. McKernan

**Affiliations:** 1 Pharmacology & Therapeutics, School of Pharmacy & Medical Sciences, University of Galway, Ireland; 2 School of Psychology, University of Galway, Ireland; 3 School of Biological and Chemical Sciences, University of Galway, Ireland; 4 School of Medicine, University of Galway, Ireland; 5 Centre for Neuroimaging, Cognition and Genomics (NICOG),University of Galway, Ireland; 6 Department of Psychiatry, Trinity College Dublin, St. James’s Hospital, Dublin, Ireland

**Keywords:** Antipsychotic drugs, Toll-like receptor, inflammation, cytokine, schizophrenia, cognition, social cognition, moderation analysis

## Abstract

**Background:**

Schizophrenia (SZ) is a debilitating psychiatric disorder where patients experience cognitive decline. Antipsychotic drugs alleviate positive symptoms but do not improve cognitive performance. We previously demonstrated that Toll-like receptors (TLRs), involved in cytokine production, can predict cognitive deficits in SZ patients. In this study, we aim to investigate the potential moderating effects of antipsychotic drugs on the associations between cytokines, TLRs, and cognition.

**Methods:**

In total, 280 participants (201 controls and 79 cases of SZ) were recruited in Ireland. Venous blood from the participants was stimulated with TLR ligands. Levels of cytokines were measured from plasma and post-blood stimulation. The participants were administered a battery of cognitive tasks using the Cambridge Neuropsychological Test Automated Battery and Wechsler Adult Intelligence Scale-IIIR. Olanzapine equivalents were calculated using the defined daily dose method.

**Results:**

The results indicate that antipsychotic drug dose does not predict TLR activity or cognition, indicating that antipsychotic drug dose does not have a direct effect on cognition or TLR activity. However, the relationship between TLR4 activity and visual learning and memory is moderated by the antipsychotic drug dose (*B* = −0.065; *p* < 0.001), where increasing doses have a decreasing impact on their relationship.

**Conclusions:**

Our data indicate that the dose of antipsychotic drugs alone cannot predict changes in cognitive performance and TLR4-activity. It also suggests that antipsychotic drug doses significantly affect TLR activity and its relationship with cognition. These effects are more pronounced on some domains than others. These findings open up new avenues for understanding the complex interplay between antipsychotic drugs, TLRs, and cognitive deficits in SZ.

## Introduction

Schizophrenia (SZ) is a complex psychiatric disorder affecting ~24 million people or 1 in 300 individuals worldwide, according to the 2021 census (WHO, 2022). Antipsychotic drugs are prescribed to such patients alone or in combination (with other antipsychotic agents) to alleviate symptoms (Doane et al., 2020; Lähteenvuo & Tiihonen, 2021). There is substantial evidence in the literature showing the immunomodulatory effect of antipsychotic drugs (Drzyzga, Obuchowicz, Marcinowska, & Herman, 2006). For example, one study reported that in female SZ patients, increasing doses of antipsychotic drugs were associated with higher neutrophil-to-lymphocyte ratio (NLR) and platelet-to-lymphocyte ratio (PLR) (Frota et al., 2023). Chen and colleagues reported suppression of interferon-γ (IFNγ) production by clozapine in peripheral blood mononuclear cells (PBMCs) (Chen et al., 2012) and noted that both risperidone and clozapine inhibited the production of interleukin-6 (IL-6), IL-8, and IL-12, and increased IL-10 production in lipopolysaccharide (LPS)-stimulated macrophages (Chen et al., 2013). Similarly, in polyinosinic:polycytidylic acid (PIC) and LPS-stimulated PBMC cultures, haloperidol, clozapine, risperidone, and quetiapine increased the production of anti-inflammatory cytokines (IL-4 and IL-10), while decreasing the production of pro-inflammatory cytokine (IFNγ) (Al-Amin, Uddin, & Reza, 2013). *In vitro* studies also show decreased neutrophil survival rate post-clozapine treatment (48 h, 50 μM clozapine) (Goto et al., 2017). It is well documented that clozapine is associated with neutropenia in 3% of cases and agranulocytosis in 0.8% of cases (Matsui et al., 2020; Mijovic & MacCabe, 2020). Furthermore, a recent meta-analysis demonstrated that risperidone had clear anti-inflammatory effects, with a decrease in IL-6, IL-1β, and tumor necrosis factor-α (TNF-α) in the plasma/serum of patients, and no effect was observed in the case of clozapine (S. R. Patlola, Donohoe, & McKernan, 2023a).

Studies have also shown the immunomodulatory effect of antipsychotic drugs on Toll-like receptors (TLRs), whose activity regulates the production of cytokines. One particular study on human postmortem brain determined that the patients on antipsychotic drugs showed higher protein expression of TLR4 and MyD88 compared to controls, and only for MyD88, messenger RNA (mRNA) expression was significantly higher in the antipsychotic drug group compared to the drug-naïve SZ group (García-Bueno et al., 2016). Other studies have reported that the percentage of TLR4^+^ and TLR5^+^ monocyte cells in blood decreased in patients after risperidone or olanzapine (OLZ) treatment (8 weeks), whereas the percentage of TLR2^+^ monocytes increased (Kéri, Szabó, & Kelemen, 2017). Balaji and colleagues reported no change in mRNA expression levels of TLR3 and 4 in PBMCs after 3 months of antipsychotic medication (Balaji et al., 2020). At the cellular level, antipsychotic drugs seem to contribute toward the changes in immune cell counts, while at the molecular level, they seem to affect the TLR number, activity, and their downstream signaling products, such as cytokines.

Immune modulation by antipsychotic drugs has been associated with changes in cognitive function. For example, Kéri et al. (2017) reported that higher percentages of TLR4^+^ and TLR5^+^ monocyte levels were found to be associated with decreased cognitive performance, which was not observed post-antipsychotic drug administration (Kéri et al., 2017). Frota et al. reported that higher antipsychotic drug doses were associated with performance decline in working memory, processing speed (PS), and executive function, possibly due to changes in the immune system (NLR and PLR ratios) (Frota et al., 2023). Furthermore, a recent article discusses the cognitive benefits of reducing the antipsychotic drug dose. They also discuss the effects of antipsychotic drugs on neuroinflammation, further modulating neurotransmitter pathways and cognition (Allott et al., 2024). Consequently, based on current research, antipsychotic drugs might have an immunomodulatory effect and negatively affect cognitive performance. However, it is unclear if immune alteration mediates the cognitive changes due to antipsychotic drugs. Moreover, our recent work showed that TLR2 and TLR4 activity negatively impact cognition via cytokines (Patlola et al., 2025). Hence, we are interested in understanding if the changes in the immune system and cognition are affected by antipsychotic drugs.

Another crucial factor that needs to be taken into account is the anticholinergic burden (ACB). Most drugs used to treat psychiatric disorders, including antipsychotic drugs, mood stabilizers, and antidepressants, tend to have anticholinergic side effects (Lieberman 3rd, 2004) and can cause cognitive impairment. Past studies have demonstrated that ACB has a significant impact on the functional capacity and cognition of individuals with SZ (Joshi et al., 2021; O’Reilly et al., 2016). This effect was shown to be more pronounced in verbal memory, mediating the worsening of functioning in first-episode psychosis cohort (Ballesteros et al., 2021). Therefore, we incorporated both antipsychotic drug dose and ACB in our analysis.

In this study, we hypothesized that antipsychotic drugs have an immunomodulatory effect on cytokine levels and TLR activity. We also aim to investigate whether antipsychotic medication affects cognition and whether the antipsychotic drug dose moderates the relationship between immune biomarkers and cognition.

## Methods and materials

### Participants

From a total of 300 participants recruited as part of the ‘Immune Response & Social Cognition in Schizophrenia’ research project, 280 (*N* = 79 patients and 201 healthy controls [HCs]) had the necessary data available for this study. This is a cross-sectional and observational study design. The patient group consisted of clinically stable patients with either a diagnosis of SZ or schizoaffective disorder. Diagnosis was confirmed using the Structured Clinical Interview for Diagnostic Statistical Manual-IV, and patients were recruited from local outpatient clinics and mental health services. The Positive and Negative Syndrome Scale (PANSS) was used to measure the symptoms and severity of SZ, with the Hamilton Depression and Rating Scale (HAM-D) utilized to measure depressive symptoms. Both the PANSS and HAM-D (17-item) have high reliability and validity metrics (Kay, Fiszbein, & Opler, 1987; Maier & Philipp, 1985). Healthy participants were recruited from the general population. Participants were excluded based on the following criteria: (1) a history of acquired brain injury causing loss of consciousness of >1 min; (2) substance abuse in the last 6 months, and (3) intellectual disability. All participants were aged between 18 and 65 years. Additional information on the sample, including full inclusion and exclusion criteria, has been detailed in the Supplementary Methods. All individuals gave informed written consent before the study, and assessments were conducted in accordance with the relevant ethics committee approval.

### Blood collection and plasma isolation

Venous blood was collected from HCs and SZ patients in 10 mL EDTA tubes (Catalog# BD367873) for plasma and 2.5 mL PAXgene® Blood tubes for RNA (Qiagen, Catalog# 762165) at ~9.00 AM (for every participant). Approximately 3–4 mL of blood (EDTA tubes) was centrifuged at 1,200 g for 10 min at room temperature, and the resultant supernatant (plasma) was then aliquoted and stored at −80 °C for further analysis. The remaining blood was used for stimulation studies carried out immediately after collection. Additional information is available in the Supplementary Methods.

### Immune assays for protein quantification

Enzyme-linked immunosorbent assay (ELISA) was used to quantify cytokine and C-reactive protein (CRP) levels in blood plasma and from stimulated whole blood cultures. For stimulations, TLR ligands were used, such as TLR2 – heat-killed *Listeria monocytogenes* (10^10^ cells of HKLM) (Invivogen, Catalog# tlrl-hklm), TLR3 – 10 μg/mL PIC (Invivogen, Catalog# tlrl-pic), and TLR4–1 μg/mL LPS (Invivogen, Catalog# tlrl-eklps). Blood cultures were treated with the ligands for 24 h, and the supernatant was harvested. This was stored at −80 °C for later analysis. Cytokines, such as IL-6, IL-8, IL-10, and TNF-α, were assessed using the supernatant by DuoSet ELISA kits (Bio-Techne Ltd.; R&D Systems) and plasma cytokines by Quantikine High Sensitivity ELISA kits (and Quantikine kit for CRP) from Bio-Techne Ltd. The procedures and technical information for both assays are detailed in the Supplementary Methods.

### Cognitive assessment

The participant’s cognitive performance was recorded using Wechsler scales and Cambridge Neuropsychological Test Automated Battery (CANTAB) tests. The tasks measured and utilized in this study include digit symbol coding (scores 0–133), logical memory-recall (scores 0–75; 25/iteration), full-scale intelligence quotient, letter-number sequence (21 items; scores 0–21), paired associates learning (total errors scores from 6 shapes adjusted), and reading in the mind of the eyes task (18 items; 0–18). Furthermore, these cognition tasks were assigned to specific domains (Supplementary Methods and Supplementary Table S1).

### OLZ dose equivalents calculation

The dose from each antipsychotic drug was converted to the equivalent of OLZ based on the defined daily dose method from the World Health Organization, calculated by Leucht, Samara, Heres, & Davis (2016). In the case of polypharmacy (multiple antipsychotic drugs prescribed in combination) or parenteral administrations, drug doses per day were calculated, which were then converted to OLZ equivalents. These antipsychotic drug daily doses were added to get the total OLZ equivalent dose/day/individual.

### ACB scores calculation

ACB scores were calculated based on a scale from 0 to 3, with 0 being no anticholinergic effect, a score of 1 indicating a possible anticholinergic effect, and scores of 2 and 3 indicating a definite anticholinergic effect (Fox et al., 2011). Using an online tool (King Rebecca, 2024), ACB scores were calculated for all drugs, including antipsychotic drugs, and psychiatric and nonpsychiatric drugs. Individual drug ACB scores were summed for every participant to get a cumulative ACB score, which is used in the analysis.

### Statistical analysis

The data were tested for normality using the Shapiro–Wilk test and homogeneity of variance using Fisher’s test. Plasma cytokines, TLR activity, and cognition were all analyzed using Mann–Whitney unpaired *t*-test using GraphPad Prism V.10.3.1. Bonferroni correction was applied to these tests to factor for multiple testing. Multiple linear regressions, principal component analysis (PCA), and moderation analysis were conducted using IBM SPSS V.29 (IBMCorp, 2023). Bootstrapping was performed in these analyses to address the disproportionate sample variances between controls and patients, along with increasing the confidence of our results. Sample size variations in linear regression data reflect the missing data either in the dependent or independent variables, or both.

Model summaries, sample sizes, and analysis of variance were used to check the quality of multiple linear regressions. For multiple linear regressions, the Kaiser–Meyer–Olkin measure of sampling adequacy, Bartlett’s test of sphericity, and scree plots were used as quality control.

We used moderation analysis in our study, and this investigates if the relationship between two variables is strengthened or weakened depending on the level of the third variable knowns as a moderator. Moderation analysis was conducted using PROCESS V.4.2 in SPSS. ‘Model 1’ (Stride, Gardner, Catley, & Thomas, 2015) was used in our study, and its quality was determined using model summaries. TLR2 and 4 activities are composite variables based on the PCA of IL-6, IL-8, IL-10, and TNF-α levels from blood stimulations (data presented in Patlola et al. [2025]). The detailed list of quality checks and model summaries is available in the Supplementary Documents.

## Results

### Sociodemographic data

The detailed sociodemographic data are presented in Table 1. Patients (SZ) in our study were older (*p* < 0.0001) and spent a similar time in education (*p* = 0.43) compared to HCs. Individuals with SZ had a higher body mass index (BMI) (*p* < 0.0001) and showed a higher incidence of depression (*p* < 0.0001). Patients also showed significantly lower cognitive performance (*p* < 0.05) than controls in full-scale IQ, digit symbol coding, logical memory, letter-number sequence, paired associates learning, and reading of mind in the eyes tasks. The results shown here belong to subset of full cohort (Patlola et al., 2025). The subset was filtered to remove patients not taking any medication or presented incomplete information on medication. Participants were also excluded if they had high CRP levels (>10 mg/L), as it could be due to an underlying health condition that was not known to the participant at that time or was not reported.

**Table 1.** Sociodemographic and cognition data

	Healthy controls (HCs) (*N* = 201)		Schizophrenia patients (SZ) (*N* = 79)		HC vs. SZ
	Mean	Std. Dev.	Mean	Std. Dev.	*P* value
Sex (M/F)	117/84		53/26		0.09
Age (years)	36.08	12.2	42.53	10.83	**<0.0001**
BMI	24.55	3.71	29.74	4.99	**<0.0001**
Education (years)	14	7.08	13.9	5.11	0.43
Illness duration (years)	“	–	16.28	10.52	–
*PANSS*					
Positive score	–	–	8.6	2.6	–
Negative score	–	–	9.3	3.36	–
General score	–	–	20.9	4.24	–
Total score	–	–	38.8	8.47	–
HAM-D 17	2.6	3.4	4.9	4.34	**<0.0001**
Antipsychotic dose (mg/day) (OLZ equiv.)	–	–	17.79	24.56	–
Anticholinergic burden (ACB)	0.01	0.1	3.66	2.2	**<0.001**
*Cognition*					
Full-scale IQ	112.33	16.12	94.32	16.7	**<0.0001**
DSC	76.83	15.04	55.53	16.59	**<0.0001**
LM (recall)	45.59	10.04	33.35	11.43	**<0.0001**
LNS	11.16	2.68	8.21	3.12	**<0.0001**
PAL	2.28	3.3	7.81	7.74	**<0.0001**
RME	27.36	4.21	22.85	4.98	**<0.0001**

*Note: P*-values in this table are based on a non-parametric (Mann–Whitney test) *T*-test between HC and SZ. BMI, body mass index; PANSS, Positive and Negative Symptom Scale; HAM-D 17, Hamilton Depression Rating Scale 17 items; antipsychotic drug, antipsychotic drug; OLZ, olanzapine; IQ, intelligence quotient; DSC, digit symbol coding; LM, logical memory; PAL, paired associates learning; LNS, letter number sequencing; RME, reading the mind in the eyes. *N*, sample size; Std. Dev., standard deviation; Bold, significant.

### TLR activity and plasma cytokines

TLR activity is a composite score measure derived from a PCA based on the levels of stimulated cytokine release (IL-6, IL-8, IL-10, and TNF-α) following TLR activation for 24 h using agonists specific for each TLR, for example, TLR2 – HKLM (10^10^ cells), TLR3 – Poly I:C (10 μg/mL), and TLR4 – LPS (1 μg/mL). Participants in the SZ cohort displayed higher mean TLR2 and four activities (*p* < 0.01) compared to HCs (Table 2). Patients showed significant elevation in circulating cytokines (excluding IL-10, IL-12, and IFN-γ; *p* > 0.05) compared to HCs. We have reported this for the full cohort, but as this is a subset, we are reporting the new values (Patlola et al., 2025). Only TLR2, TLR4, and IL-8 survived the Bonferroni multiple-test correction.

**Table 2.** TLR2 and 4 activity and cytokine data

	Healthy controls (HCs) (*N* = 201)		Schizophrenia patients (SZ) (*N* = 79)		HC vs. SZ
	Mean	Std. Dev.	Mean	Std. Dev.	*P* value
*TLR activity*					
TLR2	−0.15	0.87	0.24	1.05	**0.006**
TLR4	−0.21	0.63	0.37	1.37	**0.002**
*Plasma cytokines*					
IL6 (pg/mL)	1.48	1.59	3.01	5.14	**0.013**
IL8 (pg/mL)	3.79	2.35	5.54	3.68	**<0.0001**
TNF-α (pg/mL)	0.92	0.38	1.08	0.46	**0.011**
IL10 (pg/mL)	0.21	0.27	0.95	4.89	0.251
IL12 (pg/mL)	0.39	0.26	0.44	0.42	0.411
IFN-γ (pg/mL)	0.61	1.84	0.46	0.54	0.357

*Note: P* values in this table are based on a non-parametric (Mann–Whitney test) *t*-test between HCs and patients. TLR, Toll-like receptor; IL, interleukin; TNF, tumor necrosis factor; IFN, interferon; pg/ml, picograms per milli-liter; *N*, sample size; Std. Dev., standard deviation; Bold, significant.

### Antipsychotic drugs/OLZ equivalent doses and other medications

**Figure 1.**
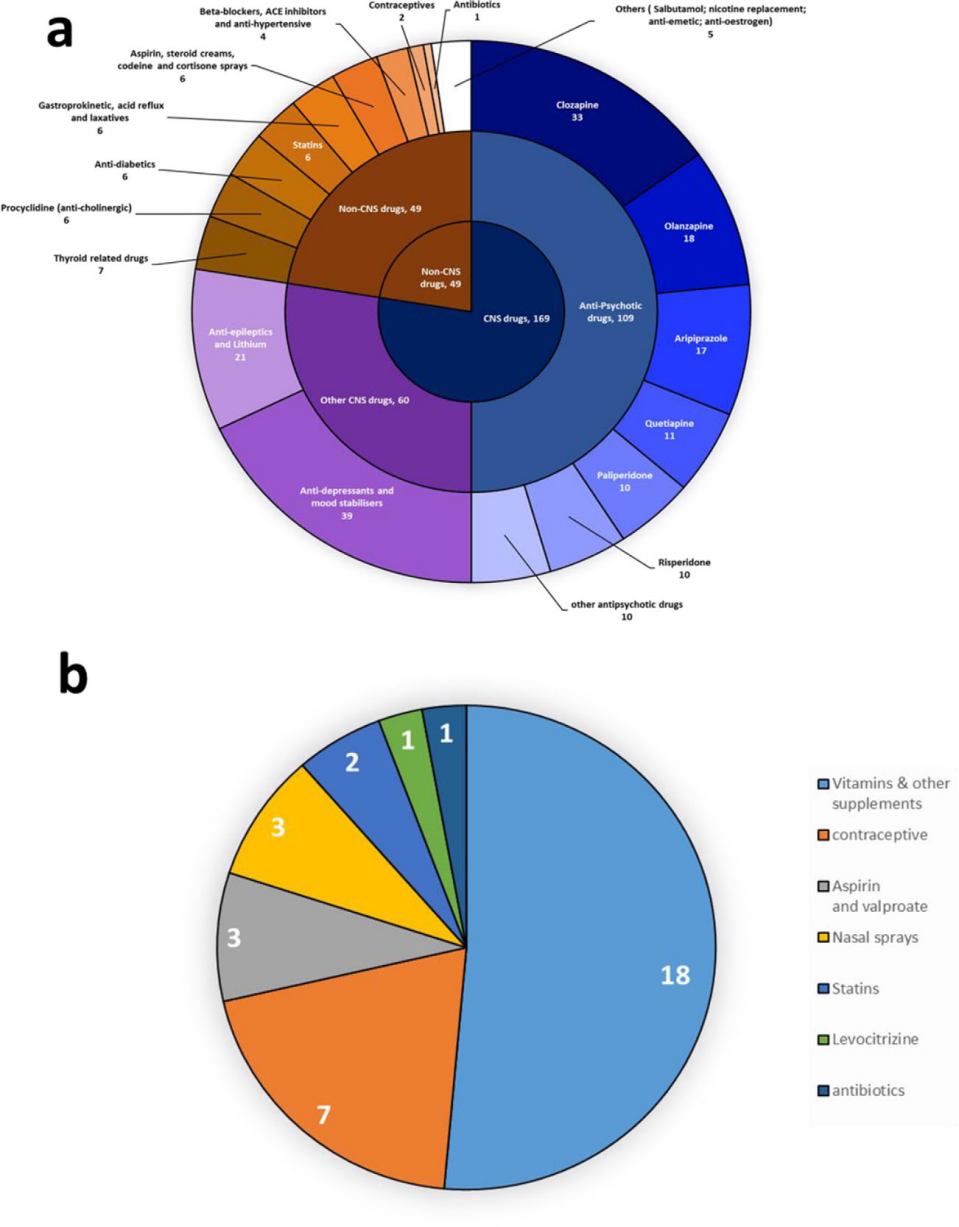
Breakdown of all the medication taken by patients. (a) Doughnut chart; *N* = 79 and controls. (b) Pie chart; *N* = 25. The number below the drug(s) indicates the number of participants taking that medication at the time of the study. CNS, central nervous system; ACE, acetylcholinesterase.

After calculating the OLZ equivalents, the participants were categorized based on the number of antipsychotic drugs they were taking at the time of study (Supplementary Table S2). Fifty-four patients are taking one antipsychotic drug, and the rest are on multiple antipsychotic drugs (22 SZ – 2 antipsychotic drugs; 3 SZ – 3 antipsychotic drugs). Patients with no antipsychotic drugs or incomplete information were excluded from further analysis. Other medications acting on the central nervous system (CNS) and non-CNS drugs were categorized separately. Only 25 HCs were on some form of medication or supplements. The medication details for both patients and controls are illustrated in Figure 1.

### Antipsychotic drug dose does not predict the decline in cognition

Multiple linear regression was performed on the whole population using antipsychotic drug dose as the independent variable and cognition as the dependent variable, controlling for age, sex, BMI, full-scale IQ, and duration of illness (Table 3). This analysis showed that antipsychotic drug dose does not have any significant association with cognition in our cohort. An increase in duration of illness, age, and BMI (in some cases) significantly predicted a decline in cognition (*p* < 0.05) when the remaining variables were kept constant. Additionally, a greater IQ was associated with increased cognitive performance when the rest of the variables were kept constant. Model summary and specifics are detailed in Supplementary Doc 1.

**Table 3.** Multiple linear regression of cognition, TLR activity, and cytokines

Dependent variable (sample size)	Constant	Antipsychotic drug dose (olanzapine equivalents) (mg/day)	FSIQ	Duration of illness (years)	Sex (0 female, 1 male)	Age (years)	BMI
Cognition							
PS (264)	46.781***	−0.047	0.467***	−0.323**	−4.223**	−0.39***	−0.263
VLM (258)	27.028***	0.005	0.208***	−0.227*	−0.739	−0.094	−0.086
VisLM (265)	8.689**	−0.022	−0.079***	0.24***	−0.257	0.079**	−0.009
WM (244)	4.89**	0.006	0.053***	−0.102***	0.113	0.044**	−0.06
SC (265)	21.535***	−0.037	0.091***	−0.038	−0.069	0.007	−0.196**
TLR activity and cytokine							
TLR2 (243)	−0.399	−0.002	6.33E–05	0.023**	0.038	0.003	0.005
TLR4 (181)	−0.198	0.008	−0.003	0.005	0.035	0	0.016
IL–8 (253)	9.26***	0.001	−0.031**	0.068**	0.238	−0.001	−0.078

*Note:* Sample size variations in the data reflect the missing data either in the dependent or independent variables or both. FSIQ, full-scale intelligence quotient; PS, processing speed; VLM, verbal learning and memory; VisLM, visual learning and memory; WM, working memory; SC, social cognition; TLR, Toll-like receptors; IL, interleukin; BMI, body mass index; *p*, *p*-value; Bold, significant relationships.

### Antipsychotic drug dose does not predict changes in TLR activity or cytokines

Multiple linear regression was performed in the whole population using TLR activity and IL-8 (only cytokine to survive Bonferroni correction) (Table 3) as dependent variables and antipsychotic drug dose as an independent variable, factoring in age, sex, duration of illness, and BMI. We found that antipsychotic drug dose could not predict changes in TLR2/TLR4 activity and IL-8 levels in the participants. However, an increase in IQ was associated with a decrease in plasma IL-8 levels. Model summary and specifics are detailed in Supplementary Doc 1.

### Antipsychotic drug doses moderate the relationship between TLR4 activity and cognition

**Figure 2.**
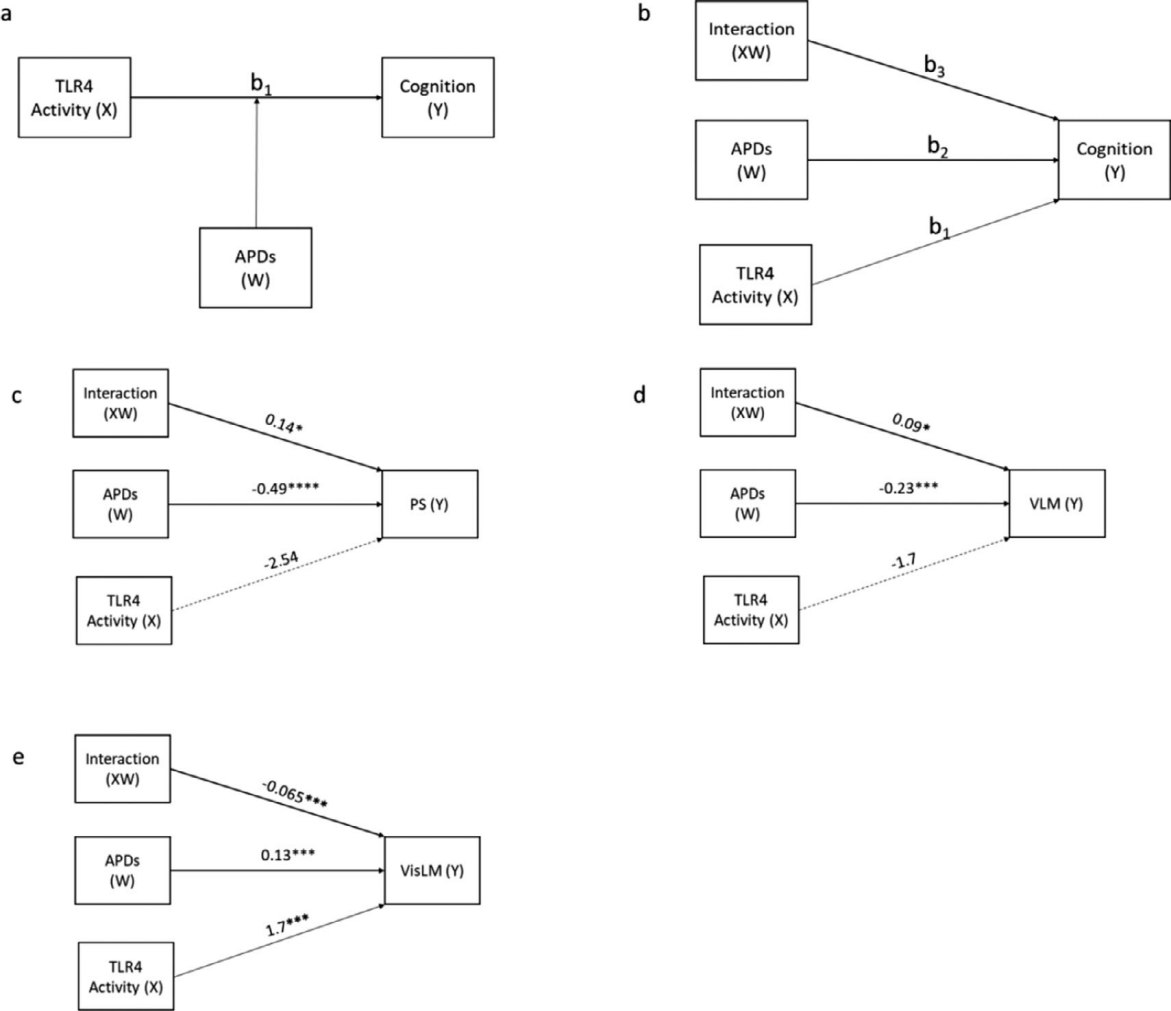
Moderation ‘Model 1’ illustrates (a) the general model and (b) the statistical model. The moderating effect of antipsychotic drug dose (APD) on the relationship between TLR4 activity and (c) processing speed (PS), (d) verbal learning and memory, and (e) visual learning and memory. TLR, Toll-like receptor; antipsychotic drug, antipsychotic drug dose; X, independent variable; Y, dependent variable; W, – moderator; path b_1_, direct effect of TLR4 activity on cognition scores; path b_2_, direct effect of antipsychotic drug dose on activity on cognition scores; path b_3_, interaction term, effect of TLR4 activity on cognition when antipsychotic drug dose is factored. *P* > 0.05, not significant; *P < 0.05, **P < 0.01, ***P < 0.001, and ****P < 0.0001.

We had previously investigated and established the mediating effects of cytokines between TLR activity and cognition in the same cohort (Patlola et al., 2025). Therefore, in the current study, we investigated whether the antipsychotic drug dose moderates (Figure 2) the relationship between immune response (inflammation) and cognition. Therefore, we investigated the moderating effect of antipsychotic drug doses on the relationship between TLR activity and cognition. The results indicate that there is no moderating effect of antipsychotic drug dose on the relationship between TLR2 activity and any of the six cognition domains. The detailed moderation analysis data and model statistics are present in Supplementary Doc 2.

However, in the case of TLR4 activity, we observed three models that showed significant associations. The results indicate that an increase in the antipsychotic drug dose strengthens the relationship between TLR4 activity and PS (*B* = 0.14; *p* < 0.05) (Figure 2c) and TLR4 activity and verbal learning and memory (VLM) (*B* = 0.09; *p* < 0.05) (Figure 2d). However, post hoc analysis shows that the conditional effects (Supplementary Figures S1 and S2) at low, moderate, and high doses are not significant, indicating that although the moderation effect is statistically significant, the effect of TLR4 activity on either PS or VLM is weak to negligible. Interestingly, we found that the antipsychotic drug dose significantly weakens the relationship between TLR4 activity and visual learning and memory (VisLM) (Figure 2e). Further post hoc analysis (conditional effects) (Supplementary Figure S3) revealed that, at low/no doses of antipsychotic drugs, an increase in TLR4 activity leads to increased scores (PAL error scores) in the VisLM domain, indicating greater cognitive deficits. The strength of this relationship decreases with an increase in dose, and finally, at high doses, the moderating effect becomes negligible.

Bonferroni multiple test correction *p*-value is set at *p* < 0.0083 for these analyses (0.02/6). TLR4/VisLM is the only model that survived the correction. All of the abovementioned models with TLR2, TLR4 activity, and cognition are not significant when covaried for age, sex, BMI, and duration of illness.

## Discussion

The effect of antipsychotic drugs on cognition has been studied for more than two decades (Baldez et al., 2021; Keefe, Silva, Perkins, & Lieberman, 1999). Evidence suggests that antipsychotic drugs provide symptom alleviation to some extent and improve cognitive deficits (Haddad et al., 2023; Hou et al., 2020) in specific domains (case-dependent). A recent network meta-analysis indicates that individual antipsychotic drugs may not have a better outcome compared to the placebo group, but in combination with other antipsychotics show a better outcome on cognition (Feber et al., 2024). However, the exact mechanism by which they impact cognition is not clearly understood. In previous meta-analyses, we showed the suppressive effect of antipsychotic drugs on inflammatory cytokines (S. R. Patlola et al., 2023a) and also the detrimental effects of elevated cytokines on cognition (Patlola, Donohoe, & McKernan, 2023b). We have also recently determined that TLR activity may influence cognitive deficits in patients (Patlola et al., 2025). We, therefore, investigated the effect of the antipsychotic drug dose on cognition and the immune system. To the best of our knowledge, this is the first study investigating whether antipsychotic drugs moderate the relationship between TLR activity and cognition.

We first investigated whether antipsychotic drug dose predicted cognitive function. Interestingly, we found no association between antipsychotic drug doses and cognition. Our results are in contrast to studies in the literature showing improved cognition in animals (MacDowell et al., 2017; Neill et al., 2016) and humans (Noh et al., 2020) post-antipsychotic drug administration. Moreover, a past meta-analysis of 35 studies (SZ patients) (Nielsen et al., 2015) indicated that clozapine and OLZ use resulted in a decline in verbal working memory in individuals with SZ. The study also showed that clozapine and OLZ improved verbal fluency. Furthermore, it was observed that first-generation antipsychotics and clozapine may have detrimental effects on cognition (Feber et al., 2024). In our cohort, 41.8% and 22.8% of patients are on clozapine and OLZ, respectively; nearly one-third of the patients (31.7%) are on more than a single antipsychotic drug. This makes it difficult to assess the effect of each drug independently in our cohort. A newer study reports a decline in cognitive function in patients with SZ, considering the cumulative lifetime antipsychotic drug use by each patient (Husa et al., 2017). Furthermore, studies suggest that reducing the antipsychotic drug dose improves cognition in SZ (Kawai et al., 2006; Singh et al., 2022).

Next, we investigated whether antipsychotic drugs affect inflammatory cytokines and TLR activity. In our previous meta-analysis, we reported that antipsychotic drugs, specifically risperidone administration, reduced plasma cytokine levels (S. R. Patlola et al., 2023a). However, we did not find such evidence in plasma cytokines in this current study. In our study, there were only 12.7% patients on risperidone. Unlike all the studies included in the meta-analysis, our study design did not investigate the effects of antipsychotic drugs before and after administration; instead, we investigated whether the OLZ equivalent doses of antipsychotic drugs could help predict the circulating cytokine levels. This could be the reason for such contrasting findings.

Additionally, we report that antipsychotic drug dose could not predict TLR activity. Both TLR2 and TLR4 receptors play a vital role in the innate immune system. They belong to a family of pattern recognition receptors and can recognize molecular patterns, including those present in LPSs from Gram-negative bacteria and some polymers from Gram-positive bacteria, as well as damage-associated molecular patterns, such as hyaluronan, fibronectin, and other endogenous ligands released during cell damage or stress, further promoting cytokine production (Molteni, Gemma, & Rossetti, 2016). Moreover, in animals, stimulation of TLR4 has been associated with cognitive decline (Li et al., 2022), and knocking out this receptor improved specific cognitive domains (Connolly, Potter, Sexton, & Kohman, 2021; Fei et al., 2022). Hence, we were interested in investigating whether increased TLR activity negatively impacted cognition (which we have previously shown as a mediation analysis with both TLR2 and TLR4 – (Patlola et al., 2025) and whether the antipsychotic drug dose moderated this relationship.

Therefore, we performed a moderation analysis, and we observed that antipsychotic drug dose negatively affected cognition. Most importantly, we did not observe any moderation effect of the antipsychotic drug dose, except for the case of TLR4/VisLM (Figure 2e), which indicates that the negative effect of TLR4 activity on cognition is dependent on the dosage levels of antipsychotic drugs. Similar findings were reported in the past in an outpatient SZ group, but with IL-6 instead of TLR4 activity (Ribeiro-Santos et al., 2020). They observed that the association between higher serum IL-6 and cognitive decline depended on the antipsychotic drug dose. In the current study, post hoc analysis indicated that at low or no antipsychotic doses, an increase in TLR4 activity was associated with a decline in VisLM (Supplementary Figure S3); at moderate doses, this association weakened but remained significant. However, at higher doses, this relationship weakened further and was not significant. In the future, this trend is worth investigating with larger longitudinal cohorts.

While the findings reported are novel, this study also has limitations. First, this is a cross-sectional study; therefore, we could not capture the effects of the antipsychotic drugs over time, which would have been valuable to understanding the effect of antipsychotic drugs on the immune system and cognition as the illness progressed. Moreover, the sample size variation between patients (*N* = 79) and controls (*N* = 201) is considerable. Therefore, results need to be carefully interpreted. We incorporated bootstrapping into the analysis to increase the confidence in the results. Second, as the patient population was on a variety of antipsychotic drugs, such as clozapine, OLZ, aripiprazole, and other antipsychotic drugs, investigating individual antipsychotic drug effects would not be possible due to the low sample size. In addition to antipsychotic drugs, some patients were also on other CNS drugs, such as antidepressants and mood stabilizers that could impact the immune system and cognition. This factor needs to be acknowledged because ACB has been demonstrated to impact cognition; therefore, a separate moderated-moderation analysis using ACB scores was performed (Supplementary Doc 3). The results indicate that only at high doses of antipsychotic drugs, an increase in TLR4 activity led to improvement in the VLM domain. This was applicable for low, moderate, and high ACB scores. However, the limitation of this is that ACB scores are qualitative and do not account for the dose of the drug. Therefore, a better score or measure needs to be created to account for this.

Third, nearly 32% of our patient cohort (25) was on more than one antipsychotic drug. This is important because despite being all second-generation drugs, they have different receptor affinities and side effects. Feber and colleagues have also suggested that the varying effects of antipsychotic drugs on cognition are due to their different receptor-binding profiles. Their findings also suggest that antipsychotic drugs’ effects on cognition are drug-specific (Feber et al., 2024). Variations in pharmacodynamics are difficult to integrate into the analysis. However, it was partly managed by converting them all into OLZ equivalents. Hence, despite the small sample sizes (within-group), we tested for differences between individuals taking one (54) and more than one antipsychotic drugs (25). The results showed that patients on more than one antipsychotic drug presented higher BMI, plasma CRP levels, more significant depressive symptoms, and poorer PS (Supplementary Doc 2) compared to patients on a single antipsychotic drug. However, it is but a trend as they do not survive Bonferroni multiple test correction. This could perhaps be because people on higher dosages or a greater number of antipsychotic drugs to begin with had a more severe illness with greater levels of symptomatology that had been unresponsive to treatment (Gallego et al., 2012).

Lastly, we did not include the participants’ lifetime cannabis or tobacco exposure in our analysis. Evidence indicates that they both have a modulating effect on TLR activity (Cui Sun, Otálora-Alcaraz, Prenderville, & Downer, 2024; Semlali, Witoled, Alanazi, & Rouabhia, 2012). In our cohort, based on a self-reporting questionnaire, 80.4% of controls and 87.6% of patients never consumed cannabis in their lifetime or in the last 12 months. Similarly, 74.9% of controls and 60.5% of patients never consumed tobacco in their lifetime. The data we have are qualitative in nature, and the reliability of the quantity of cannabis and tobacco consumed from the questionnaire could be questionable, as the forms of consumption are different and measuring the exact dose for every consumption is not possible.

In conclusion, there was no evidence in this participant cohort that antipsychotic drugs moderated the impact of the immune system on cognition. However, we did find that the antipsychotic drug dose is significantly associated with cognitive decline and elevated TLR4 activity.

## Supporting information

10.1017/S0033291726103596.sm001 Patlola et al. supplementary material Patlola et al. supplementary material

## Data Availability

The data used in this study are available upon reasonable request.

## Supplementary material

The supplementary material for this article can be found at http://doi.org/10.1017/S0033291726103596.

## References

[r1] Al-Amin, M., Uddin, M. M. N., & Reza, H. M. (2013). Effects of antipsychotics on the inflammatory response system of patients with schizophrenia in peripheral blood mononuclear cell cultures. Clinical Psychopharmacology and Neuroscience, 11(3), 144–151. 10.9758/cpn.2013.11.3.144. 24465251 PMC3897763

[r2] Allott, K., Chopra, S., Rogers, J., Dauvermann, M. R., & Clark, S. R. (2024). Advancing understanding of the mechanisms of antipsychotic-associated cognitive impairment to minimise harm: A call to action. Molecular Psychiatry, 29(8), 2571–2574. 10.1038/s41380-024-02503-x. 38454078 PMC11412898

[r3] Balaji, R., Subbanna, M., Shivakumar, V., Abdul, F., Venkatasubramanian, G., & Debnath, M. (2020). Pattern of expression of toll like receptor (TLR)-3 and-4 genes in drug-naïve and antipsychotic treated patients diagnosed with schizophrenia. Psychiatry Research, 285, 112727. 31837816 10.1016/j.psychres.2019.112727

[r4] Baldez, D. P., Biazus, T. B., Rabelo-da-Ponte, F. D., Nogaro, G. P., Martins, D. S., Kunz, M., & Czepielewski, L. S. (2021). The effect of antipsychotics on the cognitive performance of individuals with psychotic disorders: Network meta-analyses of randomized controlled trials. Neuroscience & Biobehavioral Reviews, 126, 265–275. 10.1016/j.neubiorev.2021.03.028. 33812977

[r5] Ballesteros, A., Sánchez Torres, A. M., López-Ilundáin, J., Mezquida, G., Lobo, A., González-Pinto, A., Pina-Camacho, L., Corripio, I., Vieta, E., de la Serna, E., Mané, A., Bioque, M., Moreno-Izco, L., Espliego, A., Lorente-Omeñaca, R., Amoretti, S., Bernardo, M., & Cuesta, M. J. (2021). The longitudinal effect of antipsychotic burden on psychosocial functioning in first-episode psychosis patients: The role of verbal memory. Psychological Medicine, 51(12), 2044–2053. 10.1017/S003329172000080X. 32326991

[r6] Chen, M.-L., Tsai, T.-C., Wang, L.-K., Lin, Y.-Y., Tsai, Y.-M., M-C, L., & Tsai, F.-M. (2012). Clozapine inhibits Th1 cell differentiation and causes the suppression of IFN-γ production in peripheral blood mononuclear cells. Immunopharmacology and Immunotoxicology, 34(4), 686–694. 10.3109/08923973.2011.651535. 22268679

[r7] Chen, M.-L., Wu, S., Tsai, T.-C., L-K, W., & Tsai, F.-M. (2013). Regulation of macrophage immune responses by antipsychotic drugs. Immunopharmacology and Immunotoxicology, 35(5), 573–580. 10.3109/08923973.2013.828744. 23981042

[r8] Connolly, M. G., Potter, O. V., Sexton, A. R., & Kohman, R. A. (2021). Effects of Toll-like receptor 4 inhibition on spatial memory and cell proliferation in male and female adult and aged mice. Brain, Behavior, and Immunity, 97, 383–393. 10.1016/j.bbi.2021.06.008. 34343615 PMC8453097

[r9] Cui Sun, M., Otálora-Alcaraz, A., Prenderville, J. A., & Downer, E. J. (2024). Toll-like receptor signalling as a cannabinoid target. Biochemical Pharmacology, 222, 116082. 10.1016/j.bcp.2024.116082. 38438052

[r10] Doane, M. J., Sajatovic, M., Weiden, P. J., O’Sullivan, A. K., Maher, S., Bjorner, J. B., Sikora Kessler, A., Carpenter-Conlin, J., Bessonova, L., & Velligan, D. I. (2020). Antipsychotic treatment experiences of people with schizophrenia: Patient perspectives from an online survey. Patient Preference and Adherence, 14, 2043–2054. 10.2147/ppa.S270020. 33149559 PMC7604247

[r11] Drzyzga, Ł., Obuchowicz, E., Marcinowska, A., & Herman, Z. S. (2006). Cytokines in schizophrenia and the effects of antipsychotic drugs. Brain, Behavior, and Immunity, 20(6), 532–545. 10.1016/j.bbi.2006.02.002. 16580814

[r12] Feber, L., Peter, N. L., Chiocchia, V., Schneider-Thoma, J., Siafis, S., Bighelli, I., Hansen, W.-P., Lin, X., Prates-Baldez, D., Salanti, G., Keefe, R. S. E., Engel, R. R., & Leucht, S. (2024). Antipsychotic drugs and cognitive function: A systematic review and pairwise network meta-analysis. JAMA Psychiatry. 10.1001/jamapsychiatry.2024.2890. PMC11581732 39412783

[r13] Fei, X., Y-n, D., Lv, W., Ding, B., Wei, J., Wu, X., He, X., Fei, Z., & Fei, F. (2022). TLR4 deletion improves cognitive brain function and structure in aged mice. Neuroscience, 492, 1–17. 10.1016/j.neuroscience.2022.04.007. 35405301

[r14] Fox, C., Richardson, K., Maidment, I. D., Savva, G. M., Matthews, F. E., Smithard, D., Coulton, S., Katona, C., Boustani, M. A., & Brayne, C. (2011). Anticholinergic medication use and cognitive impairment in the older population: The Medical Research Council cognitive function and ageing study. Journal of the American Geriatrics Society, 59(8), 1477–1483. 10.1111/j.1532-5415.2011.03491.x. 21707557

[r15] Frota, I. J., de Oliveira, A. L. B., De Lima, D. N., Costa Filho, C. W. L., Menezes, C. E. S., Soares, M. V. R., Chaves Filho, A. J. M., Lós, D. B., Moreira, R. T. A., Viana, G. A., Campos, E. M., Vasconcelos, S. M. M., Seeman, M. V., Macêdo, D. S., & Sanders, L. L. O. (2023). Decrease in cognitive performance and increase of the neutrophil-to-lymphocyte and platelet-to-lymphocyte ratios with higher doses of antipsychotics in women with schizophrenia: A cross-sectional study. BMC Psychiatry, 23(1), 558. 10.1186/s12888-023-05050-x. 37532985 PMC10394759

[r16] Gallego, J. A., Nielsen, J., De Hert, M., Kane, J. M., & Correll, C. U. (2012). Safety and tolerability of antipsychotic polypharmacy. Expert Opinion on Drug Safety, 11(4), 527–542. 10.1517/14740338.2012.683523. 22563628 PMC3384511

[r17] García-Bueno, B., Gassó, P., MacDowell, K. S., Callado, L. F., Mas, S., Bernardo, M., Lafuente, A., Meana, J. J., & Leza, J. C. (2016). Evidence of activation of the Toll-like receptor-4 proinflammatory pathway in patients with schizophrenia. Journal of Psychiatry and Neuroscience, 41(3), E46. 10.1503/jpn.150195. 27070349 PMC4853215

[r18] Goto, A., Yoshimi, A., Nagai, T., Ukigai, M., Mouri, A., Ozaki, N., & Noda, Y. (2017). Human neutrophils show decreased survival upon long-term exposure to clozapine. Human Psychopharmacology, 32(6). 10.1002/hup.2629. 28913970

[r19] Haddad, C., Salameh, P., Sacre, H., Clément, J.-P., & Calvet, B. (2023). Effects of antipsychotic and anticholinergic medications on cognition in chronic patients with schizophrenia. BMC Psychiatry, 23(1), 61. 10.1186/s12888-023-04552-y. 36694187 PMC9872384

[r20] Hou, Y., Xie, J., Yuan, Y., Cheng, Z., Han, X., Yang, L., Yu, X., & Shi, C. (2020). Neurocognitive effects of atypical antipsychotics in patients with first-episode schizophrenia. Nordic Journal of Psychiatry, 74(8), 594–601. 10.1080/08039488.2020.1771767. 32496921

[r21] Husa, A. P., Moilanen, J., Murray, G. K., Marttila, R., Haapea, M., Rannikko, I., Barnett, J. H., Jones, P. B., Isohanni, M., Remes, A. M., Koponen, H., Miettunen, J., & Jääskeläinen, E. (2017). Lifetime antipsychotic medication and cognitive performance in schizophrenia at age 43 years in a general population birth cohort. Psychiatry Research, 247, 130–138. 10.1016/j.psychres.2016.10.085. 27888683 PMC5241225

[r22] IBM Corp. (2023). ver 29.0.2.0 Armonk. IBM Corp.

[r23] Joshi, Y. B., Thomas, M. L., Braff, D. L., Green, M. F., Gur, R. C., Gur, R. E., Nuechterlein, K. H., Stone, W. S., Greenwood, T. A., Lazzeroni, L. C., MacDonald, L. R., Molina, J. L., Nungaray, J. A., Radant, A. D., Silverman, J. M., Sprock, J., Sugar, C. A., Tsuang, D. W., Tsuang, M. T., … Light, G. A. (2021). Anticholinergic medication burden–associated cognitive impairment in schizophrenia. American Journal of Psychiatry, 178(9), 838–847. 10.1176/appi.ajp.2020.20081212. 33985348 PMC8440496

[r24] Kawai, N., Yamakawa, Y., Baba, A., Nemoto, K., Tachikawa, H., Hori, T., Asada, T., & Iidaka, T. (2006). High-dose of multiple antipsychotics and cognitive function in schizophrenia: The effect of dose-reduction. Progress in Neuro-Psychopharmacology and Biological Psychiatry, 30(6), 1009–1014. 16644082 10.1016/j.pnpbp.2006.03.013

[r25] Kay, S. R., Fiszbein, A., & Opler, L. A. (1987). The positive and negative syndrome scale (PANSS) for schizophrenia. Schizophrenia Bulletin, 13(2), 261–276. 10.1093/schbul/13.2.261. 3616518

[r26] Keefe, R. S. E., Silva, S. G., Perkins, D. O., & Lieberman, J. A. (1999). The effects of atypical antipsychotic drugs on neurocognitive impairment in schizophrenia: A review and meta-analysis. Schizophrenia Bulletin, 25(2), 201–222. 10.1093/oxfordjournals.schbul.a033374. 10416727

[r27] Kéri, S., Szabó, C., & Kelemen, O. (2017). Antipsychotics influence Toll-like receptor (TLR) expression and its relationship with cognitive functions in schizophrenia. Brain, Behavior, and Immunity, 62, 256–264. 28003154 10.1016/j.bbi.2016.12.011

[r28] King Rebecca RS (2024) ACB calculator. Available at https://www.acbcalc.com/.

[r29] Lähteenvuo, M., & Tiihonen, J. (2021). Antipsychotic polypharmacy for the Management of Schizophrenia: Evidence and recommendations. Drugs, 81(11), 1273–1284. 10.1007/s40265-021-01556-4. 34196945 PMC8318953

[r30] Leucht S, Samara M, Heres S, & Davis JM (2016) Dose equivalents for antipsychotic drugs: The DDD method. Schizophrenia Bulletin 42(Suppl 1), S90–S94. 10.1093/schbul/sbv167. 27460622 PMC4960429

[r31] Li, H., Chen, W., Gou, M., Li, W., Tong, J., Zhou, Y., Xie, T., Yu, T., Feng, W., Li, Y., Chen, S., Tian, B., Tan, S., Wang, Z., Pan, S., Li, N., Luo, X., Zhang, P., Huang, J., … Tan, Y. (2022). The relationship between TLR4/NF-κB/IL-1β signaling, cognitive impairment, and white-matter integrity in patients with stable chronic schizophrenia. Frontiers in Psychiatry, 13, 966657. 10.3389/fpsyt.2022.966657. 36051545 PMC9424630

[r32] Lieberman JA, 3rd (2004) Managing anticholinergic side effects. Prim Care Companion J Clin Psychiatry 6(Suppl 2), 20–23. 16001097 PMC487008

[r33] MacDowell, K. S., Munarriz-Cuezva, E., Caso, J. R., Madrigal, J. L. M., Zabala, A., Meana, J. J., García-Bueno, B., & Leza, J. C. (2017). Paliperidone reverts Toll-like receptor 3 signaling pathway activation and cognitive deficits in a maternal immune activation mouse model of schizophrenia. Neuropharmacology, 116, 196–207. 10.1016/j.neuropharm.2016.12.025. 28039001

[r34] Maier, W., & Philipp, M. (1985). Improving the assessment of severity of depressive states: A reduction of the Hamilton depression scale. Pharmacopsychiatry, 18(01), 114–115. 10.1055/s-2007-1017335.

[r35] Matsui, K., Ishibashi, M., Kawano, M., Oshibuchi, H., Ishigooka, J., Nishimura, K., & Inada, K. (2020). Clozapine-induced agranulocytosis in Japan: Changes in leukocyte/neutrophil counts before and after discontinuation of clozapine. Human Psychopharmacology, 35(4), e2739. 10.1002/hup.2739. 32420645

[r36] Mijovic, A., & MacCabe, J. H. (2020). Clozapine-induced agranulocytosis. Annals of Hematology, 99(11), 2477–2482. 10.1007/s00277-020-04215-y. 32815018 PMC7536144

[r37] Molteni, M., Gemma, S., & Rossetti, C. (2016). The role of Toll-like receptor 4 in infectious and noninfectious inflammation. Mediators of Inflammation, 2016, 6978936. 10.1155/2016/6978936. 27293318 PMC4887650

[r38] Neill, J. C., Grayson, B., Kiss, B., Gyertyán, I., Ferguson, P., & Adham, N. (2016). Effects of cariprazine, a novel antipsychotic, on cognitive deficit and negative symptoms in a rodent model of schizophrenia symptomatology. European Neuropsychopharmacology, 26(1), 3–14. 10.1016/j.euroneuro.2015.11.016. 26655189

[r39] Nielsen, R. E., Levander, S., Kjaersdam Telléus, G., Jensen, S. O. W., Østergaard Christensen, T., & Leucht, S. (2015). Second-generation antipsychotic effect on cognition in patients with schizophrenia—A meta-analysis of randomized clinical trials. Acta Psychiatrica Scandinavica, 131(3), 185–196. 10.1111/acps.12374. 25597383

[r40] Noh, S., Na, E., Park, S. J., Kim, S. H., Evins, A. E., & Roh, S. (2020). Effects of various antipsychotics on driving-related cognitive performance in adults with schizophrenia. Journal of Psychiatric Research, 131, 152–159. 10.1016/j.jpsychires.2020.08.029. 32971359

[r41] O’Reilly, K., O’Connell, P., Donohoe, G., Coyle, C., O’Sullivan, D., Azvee, Z., Maddock, C., Sharma, K., Sadi, H., McMahon, M., & Kennedy, H. G. (2016). Anticholinergic burden in schizophrenia and ability to benefit from psychosocial treatment programmes: A 3-year prospective cohort study. Psychological Medicine, 46(15), 3199–3211. 10.1017/S0033291716002154. 27576609

[r42] Patlola, S. R., Donohoe, G., & McKernan, D. P. (2023a). Anti-inflammatory effects of 2nd generation antipsychotics in patients with schizophrenia: A systematic review and meta-analysis. Journal of Psychiatric Research, 160, 126–136. 10.1016/j.jpsychires.2023.01.042. 36804109

[r43] Patlola, S. R., Donohoe, G., & McKernan, D. P. (2023b). The relationship between inflammatory biomarkers and cognitive dysfunction in patients with schizophrenia: A systematic review and meta-analysis. Progress in Neuro-Psychopharmacology and Biological Psychiatry, 121, 110668. 10.1016/j.pnpbp.2022.110668. 36283512

[r44] Patlola, S. R., Holleran, L., Dauvermann, M. R., Rokita, K., Laighneach, A., Hallahan, B., McManus, R., Kenyon, M., McDonald, C., Morris, D. W., Kelly, J. P., Donohoe, G., & McKernan, D. P. (2025). Investigating the relationship between Toll-like receptor activity, low-grade inflammation and cognitive deficits in schizophrenia patients – A mediation analysis. Brain, Behavior, and Immunity, 128, 529–539. 10.1016/j.bbi.2025.04.024. 40268064

[r45] Ribeiro-Santos, R., de Campos-Carli, S. M., Ferretjans, R., Teixeira-Carvalho, A., Martins-Filho, O. A., Teixeira, A. L., & Salgado, J. V. (2020). The association of cognitive performance and IL-6 levels in schizophrenia is influenced by age and antipsychotic treatment. Nordic Journal of Psychiatry, 74(3), 187–193. 10.1080/08039488.2019.1688389. 31738648

[r46] Semlali, A., Witoled, C., Alanazi, M., & Rouabhia, M. (2012). Whole cigarette smoke increased the expression of TLRs, HBDs, and proinflammory cytokines by human gingival epithelial cells through different signaling pathways. PLoS One, 7(12), e52614. 10.1371/journal.pone.0052614. 23300722 PMC3532503

[r47] Singh, A., Kumar, V., Pathak, H., Jacob, A. A., Venkatasubramanian, G., Varambally, S., & Rao, N. P. (2022). Effect of antipsychotic dose reduction on cognitive function in schizophrenia. Psychiatry Research, 308, 114383. 10.1016/j.psychres.2021.114383. 34999291

[r48] Stride C, Gardner S, Catley N, & Thomas F (2015) Mplus code for mediation, moderation and moderated mediation models (1–80). *Retrieved February 9, 2021.*

[r49] WHO (2022) Schizophrenia. Available at https://www.who.int/news-room/fact-sheets/detail/schizophrenia#:~:text=Some%20people%20with%20schizophrenia%20experience,worsening%20of%20symptoms%20over%20time.&text=Schizophrenia%20affects%20approximately%2024%20million,%25)%20among%20adults%20(2). (accessed 08 July 2024).

