# Supplementary material for: Investigating the relationship between Toll-like receptor activity, low-grade inflammation, cognitive deficits, and antipsychotic drug dose in schizophrenia patients: a moderation analysis
Source: Psychol Med. 2026 Mar 3;56:e63. doi: 10.1017/S0033291726103596 (PMC12969209; doi:10.1017/S0033291726103596)
Supplement: Patlola et al. supplementary material [file S0033291726103596sup001.zip › S0033291726103596sup001/Supplementary doc 1 regression cognition.docx]

**FSIQ**

| **Descriptive Statistics** | | | | | | |
| --- | --- | --- | --- | --- | --- | --- |
|  | | Statistic | Bootstrap^a^ | | | |
|  |  |  | Bias | Std. Error | 95% Confidence Interval | |
|  |  |  |  |  | Lower | Upper |
| FSIQ | Mean | 107.12 | -.02 | 1.11 | 104.89 | 109.35 |
|  | Std. Deviation | 18.029 | -.084 | .810 | 16.348 | 19.611 |
|  | N | 268 | 0 | 0 | 268 | 268 |
| APD_dose | Mean | 5.19046 | .00812 | .94968 | 3.55909 | 7.23371 |
|  | Std. Deviation | 15.566878 | -.502674 | 3.665646 | 8.241230 | 22.291079 |
|  | N | 268 | 0 | 0 | 268 | 268 |
| Sex | Mean | .61 | .00 | .03 | .54 | .66 |
|  | Std. Deviation | .489 | -.001 | .006 | .473 | .499 |
|  | N | 268 | 0 | 0 | 268 | 268 |
| Age | Mean | 37.96 | .03 | .73 | 36.58 | 39.45 |
|  | Std. Deviation | 12.115 | .004 | .403 | 11.328 | 12.889 |
|  | N | 268 | 0 | 0 | 268 | 268 |
| BMI | Mean | 26.04 | .00 | .29 | 25.47 | 26.60 |
|  | Std. Deviation | 4.734 | -.012 | .218 | 4.271 | 5.147 |
|  | N | 268 | 0 | 0 | 268 | 268 |
| a. Unless otherwise noted, bootstrap results are based on 1000 bootstrap samples | | | | | | |

| **Variables Entered/Removed**^a^ | | | |
| --- | --- | --- | --- |
| Model | Variables Entered | Variables Removed | Method |
| 1 | BMI, Sex, Age, APD_dose^b^ | . | Enter |
| a. Dependent Variable: FSIQ | | | |
| b. All requested variables entered. | | | |

| **Model Summary** | | | | | | | |
| --- | --- | --- | --- | --- | --- | --- | --- |
| Model | R | R Square | Adjusted R Square | Std. Error of the Estimate | Change Statistics | | |
|  |  |  |  |  | R Square Change | F Change | df1 |
| 1 | .425^a^ | .180 | .168 | 16.447 | .180 | 14.458 | 4 |

| **Model Summary** | | | |  |  |  |  |  |
| --- | --- | --- | --- | --- | --- | --- | --- | --- |
| Model | Change Statistics | | |  |  |  |  |  |
|  | df2 | Sig. F Change | |  |  |  |  |  |
| 1 | 263 | <.001 | |  |  |  |  |  |
|  |  |  |  | |  |  |  |  |

| a. Predictors: (Constant), BMI, Sex, Age, APD_dose |
| --- |

| **ANOVA**^a^ | | | | | | |
| --- | --- | --- | --- | --- | --- | --- |
| Model | | Sum of Squares | df | Mean Square | F | Sig. |
| 1 | Regression | 15643.155 | 4 | 3910.789 | 14.458 | <.001^b^ |
|  | Residual | 71141.024 | 263 | 270.498 |  |  |
|  | Total | 86784.179 | 267 |  |  |  |
| a. Dependent Variable: FSIQ | | | | | | |
| b. Predictors: (Constant), BMI, Sex, Age, APD_dose | | | | | | |

| **Coefficients**^a^ | | | | | | | |
| --- | --- | --- | --- | --- | --- | --- | --- |
| Model | | Unstandardized Coefficients | | Standardized Coefficients | t | Sig. | 95.0% Confidence Interval for B |
|  |  | B | Std. Error | Beta |  |  | Lower Bound |
| 1 | (Constant) | 142.091 | 6.243 |  | 22.758 | <.001 | 129.797 |
|  | APD_dose | -.135 | .070 | -.117 | -1.940 | .053 | -.272 |
|  | Sex | 1.039 | 2.075 | .028 | .501 | .617 | -3.046 |
|  | Age | -.381 | .088 | -.256 | -4.347 | <.001 | -.554 |
|  | BMI | -.785 | .232 | -.206 | -3.386 | <.001 | -1.241 |

| **Coefficients**^a^ | | | |  |  |  |  |  |
| --- | --- | --- | --- | --- | --- | --- | --- | --- |
| Model | | 95.0% Confidence Interval for B | |  |  |  |  |  |
|  |  | Upper Bound | |  |  |  |  |  |
| 1 | (Constant) | 154.384 | |  |  |  |  |  |
|  | APD_dose | .002 | |  |  |  |  |  |
|  | Sex | 5.124 | |  |  |  |  |  |
|  | Age | -.208 | |  |  |  |  |  |
|  | BMI | -.328 | |  |  |  |  |  |
|  |  |  |  | |  |  |  |  |

| a. Dependent Variable: FSIQ |
| --- |

**Attention and processing speed**

| **Descriptive Statistics** | | | | | | |
| --- | --- | --- | --- | --- | --- | --- |
|  | | Statistic | Bootstrap^a^ | | | |
|  |  |  | Bias | Std. Error | 95% Confidence Interval | |
|  |  |  |  |  | Lower | Upper |
| DSC | Mean | 70.91 | .05 | 1.15 | 68.70 | 73.29 |
|  | Std. Deviation | 18.239 | -.055 | .750 | 16.780 | 19.586 |
|  | N | 267 | 0 | 0 | 267 | 267 |
| APD_dose | Mean | 5.19117 | .01732 | .97388 | 3.55019 | 7.35201 |
|  | Std. Deviation | 15.596107 | -.351686 | 3.630894 | 8.664225 | 22.685089 |
|  | N | 267 | 0 | 0 | 267 | 267 |
| Sex | Mean | .61 | .00 | .03 | .55 | .67 |
|  | Std. Deviation | .489 | -.001 | .007 | .472 | .498 |
|  | N | 267 | 0 | 0 | 267 | 267 |
| Age | Mean | 37.91 | .00 | .73 | 36.47 | 39.27 |
|  | Std. Deviation | 12.103 | -.023 | .403 | 11.280 | 12.880 |
|  | N | 267 | 0 | 0 | 267 | 267 |
| BMI | Mean | 26.05 | -.01 | .29 | 25.49 | 26.62 |
|  | Std. Deviation | 4.739 | -.016 | .214 | 4.310 | 5.151 |
|  | N | 267 | 0 | 0 | 267 | 267 |
| a. Unless otherwise noted, bootstrap results are based on 1000 bootstrap samples | | | | | | |

| **Variables Entered/Removed**^a^ | | | |
| --- | --- | --- | --- |
| Model | Variables Entered | Variables Removed | Method |
| 1 | BMI, Sex, Age, APD_dose^b^ | . | Enter |
| a. Dependent Variable: DSC | | | |
| b. All requested variables entered. | | | |

| **Model Summary** | | | | | | | |
| --- | --- | --- | --- | --- | --- | --- | --- |
| Model | R | R Square | Adjusted R Square | Std. Error of the Estimate | Change Statistics | | |
|  |  |  |  |  | R Square Change | F Change | df1 |
| 1 | .595^a^ | .355 | .345 | 14.765 | .355 | 35.982 | 4 |

| **Model Summary** | | | |  |  |  |  |  |
| --- | --- | --- | --- | --- | --- | --- | --- | --- |
| Model | Change Statistics | | |  |  |  |  |  |
|  | df2 | Sig. F Change | |  |  |  |  |  |
| 1 | 262 | <.001 | |  |  |  |  |  |
|  |  |  |  | |  |  |  |  |

| a. Predictors: (Constant), BMI, Sex, Age, APD_dose |
| --- |

| **ANOVA**^a^ | | | | | | |
| --- | --- | --- | --- | --- | --- | --- |
| Model | | Sum of Squares | df | Mean Square | F | Sig. |
| 1 | Regression | 31375.055 | 4 | 7843.764 | 35.982 | <.001^b^ |
|  | Residual | 57113.604 | 262 | 217.991 |  |  |
|  | Total | 88488.659 | 266 |  |  |  |
| a. Dependent Variable: DSC | | | | | | |
| b. Predictors: (Constant), BMI, Sex, Age, APD_dose | | | | | | |

| **Coefficients**^a^ | | | | | | | |
| --- | --- | --- | --- | --- | --- | --- | --- |
| Model | | Unstandardized Coefficients | | Standardized Coefficients | t | Sig. | 95.0% Confidence Interval for B |
|  |  | B | Std. Error | Beta |  |  | Lower Bound |
| 1 | (Constant) | 119.668 | 5.607 |  | 21.343 | <.001 | 108.627 |
|  | APD_dose | -.182 | .062 | -.155 | -2.910 | .004 | -.305 |
|  | Sex | -4.060 | 1.867 | -.109 | -2.175 | .031 | -7.735 |
|  | Age | -.612 | .079 | -.406 | -7.749 | <.001 | -.768 |
|  | BMI | -.850 | .208 | -.221 | -4.078 | <.001 | -1.260 |

| **Coefficients**^a^ | | | |  |  |  |  |  |
| --- | --- | --- | --- | --- | --- | --- | --- | --- |
| Model | | 95.0% Confidence Interval for B | |  |  |  |  |  |
|  |  | Upper Bound | |  |  |  |  |  |
| 1 | (Constant) | 130.708 | |  |  |  |  |  |
|  | APD_dose | -.059 | |  |  |  |  |  |
|  | Sex | -.385 | |  |  |  |  |  |
|  | Age | -.457 | |  |  |  |  |  |
|  | BMI | -.439 | |  |  |  |  |  |
|  |  |  |  | |  |  |  |  |

| a. Dependent Variable: DSC |
| --- |

**Verbal learning and memory**

| **Descriptive Statistics** | | | | | | |
| --- | --- | --- | --- | --- | --- | --- |
|  | | Statistic | Bootstrap^a^ | | | |
|  |  |  | Bias | Std. Error | 95% Confidence Interval | |
|  |  |  |  |  | Lower | Upper |
| WMS | Mean | 42.15 | .02 | .75 | 40.69 | 43.68 |
|  | Std. Deviation | 11.687 | -.036 | .474 | 10.724 | 12.570 |
|  | N | 260 | 0 | 0 | 260 | 260 |
| APD_dose | Mean | 4.63623 | -.02001 | .94403 | 2.91998 | 6.67265 |
|  | Std. Deviation | 15.065219 | -.725820 | 3.917117 | 6.936095 | 22.013549 |
|  | N | 260 | 0 | 0 | 260 | 260 |
| Sex | Mean | .61 | .00 | .03 | .55 | .67 |
|  | Std. Deviation | .489 | -.001 | .007 | .473 | .499 |
|  | N | 260 | 0 | 0 | 260 | 260 |
| Age | Mean | 37.85 | .03 | .77 | 36.38 | 39.39 |
|  | Std. Deviation | 12.123 | -.022 | .388 | 11.318 | 12.903 |
|  | N | 260 | 0 | 0 | 260 | 260 |
| BMI | Mean | 25.94 | .00 | .30 | 25.37 | 26.51 |
|  | Std. Deviation | 4.711 | -.015 | .218 | 4.256 | 5.106 |
|  | N | 260 | 0 | 0 | 260 | 260 |
| a. Unless otherwise noted, bootstrap results are based on 1000 bootstrap samples | | | | | | |

| **Variables Entered/Removed**^a^ | | | |
| --- | --- | --- | --- |
| Model | Variables Entered | Variables Removed | Method |
| 1 | BMI, Sex, Age, APD_dose^b^ | . | Enter |
| a. Dependent Variable: WMS | | | |
| b. All requested variables entered. | | | |

| **Model Summary** | | | | | | | |
| --- | --- | --- | --- | --- | --- | --- | --- |
| Model | R | R Square | Adjusted R Square | Std. Error of the Estimate | Change Statistics | | |
|  |  |  |  |  | R Square Change | F Change | df1 |
| 1 | .344^a^ | .118 | .104 | 11.060 | .118 | 8.556 | 4 |

| **Model Summary** | | | |  |  |  |  |  |
| --- | --- | --- | --- | --- | --- | --- | --- | --- |
| Model | Change Statistics | | |  |  |  |  |  |
|  | df2 | Sig. F Change | |  |  |  |  |  |
| 1 | 255 | <.001 | |  |  |  |  |  |
|  |  |  |  | |  |  |  |  |

| a. Predictors: (Constant), BMI, Sex, Age, APD_dose |
| --- |

| **ANOVA**^a^ | | | | | | |
| --- | --- | --- | --- | --- | --- | --- |
| Model | | Sum of Squares | df | Mean Square | F | Sig. |
| 1 | Regression | 4186.095 | 4 | 1046.524 | 8.556 | <.001^b^ |
|  | Residual | 31191.751 | 255 | 122.321 |  |  |
|  | Total | 35377.846 | 259 |  |  |  |
| a. Dependent Variable: WMS | | | | | | |
| b. Predictors: (Constant), BMI, Sex, Age, APD_dose | | | | | | |

| **Coefficients**^a^ | | | | | | | |
| --- | --- | --- | --- | --- | --- | --- | --- |
| Model | | Unstandardized Coefficients | | Standardized Coefficients | t | Sig. | 95.0% Confidence Interval for B |
|  |  | B | Std. Error | Beta |  |  | Lower Bound |
| 1 | (Constant) | 60.411 | 4.260 |  | 14.180 | <.001 | 52.021 |
|  | APD_dose | -.073 | .049 | -.094 | -1.492 | .137 | -.170 |
|  | Sex | -.587 | 1.414 | -.025 | -.415 | .679 | -3.372 |
|  | Age | -.216 | .060 | -.224 | -3.613 | <.001 | -.334 |
|  | BMI | -.362 | .159 | -.146 | -2.274 | .024 | -.676 |

| **Coefficients**^a^ | | | |  |  |  |  |  |
| --- | --- | --- | --- | --- | --- | --- | --- | --- |
| Model | | 95.0% Confidence Interval for B | |  |  |  |  |  |
|  |  | Upper Bound | |  |  |  |  |  |
| 1 | (Constant) | 68.801 | |  |  |  |  |  |
|  | APD_dose | .023 | |  |  |  |  |  |
|  | Sex | 2.198 | |  |  |  |  |  |
|  | Age | -.098 | |  |  |  |  |  |
|  | BMI | -.048 | |  |  |  |  |  |
|  |  |  |  | |  |  |  |  |

| a. Dependent Variable: WMS |
| --- |

**Working memory**

| **Descriptive Statistics** | | | | | | |
| --- | --- | --- | --- | --- | --- | --- |
|  | | Statistic | Bootstrap^a^ | | | |
|  |  |  | Bias | Std. Error | 95% Confidence Interval | |
|  |  |  |  |  | Lower | Upper |
| LNS | Mean | 10.36 | -.01 | .20 | 9.98 | 10.75 |
|  | Std. Deviation | 3.088 | -.007 | .137 | 2.811 | 3.343 |
|  | N | 246 | 0 | 0 | 246 | 246 |
| APD_dose | Mean | 4.69847 | -.02294 | .99593 | 3.01303 | 6.88279 |
|  | Std. Deviation | 15.606211 | -.582236 | 3.968759 | 7.671432 | 23.046083 |
|  | N | 246 | 0 | 0 | 246 | 246 |
| Sex | Mean | .61 | .00 | .03 | .54 | .66 |
|  | Std. Deviation | .490 | -.001 | .007 | .475 | .499 |
|  | N | 246 | 0 | 0 | 246 | 246 |
| Age | Mean | 37.57 | -.03 | .82 | 35.93 | 39.19 |
|  | Std. Deviation | 12.203 | -.050 | .422 | 11.273 | 12.913 |
|  | N | 246 | 0 | 0 | 246 | 246 |
| BMI | Mean | 25.89 | -.01 | .30 | 25.30 | 26.47 |
|  | Std. Deviation | 4.602 | -.015 | .216 | 4.154 | 5.010 |
|  | N | 246 | 0 | 0 | 246 | 246 |
| a. Unless otherwise noted, bootstrap results are based on 1000 bootstrap samples | | | | | | |

| **Variables Entered/Removed**^a^ | | | |
| --- | --- | --- | --- |
| Model | Variables Entered | Variables Removed | Method |
| 1 | BMI, Sex, Age, APD_dose^b^ | . | Enter |
| a. Dependent Variable: LNS | | | |
| b. All requested variables entered. | | | |

| **Model Summary** | | | | | | | |
| --- | --- | --- | --- | --- | --- | --- | --- |
| Model | R | R Square | Adjusted R Square | Std. Error of the Estimate | Change Statistics | | |
|  |  |  |  |  | R Square Change | F Change | df1 |
| 1 | .287^a^ | .082 | .067 | 2.983 | .082 | 5.388 | 4 |

| **Model Summary** | | | |  |  |  |  |  |
| --- | --- | --- | --- | --- | --- | --- | --- | --- |
| Model | Change Statistics | | |  |  |  |  |  |
|  | df2 | Sig. F Change | |  |  |  |  |  |
| 1 | 241 | <.001 | |  |  |  |  |  |
|  |  |  |  | |  |  |  |  |

| a. Predictors: (Constant), BMI, Sex, Age, APD_dose |
| --- |

| **ANOVA**^a^ | | | | | | |
| --- | --- | --- | --- | --- | --- | --- |
| Model | | Sum of Squares | df | Mean Square | F | Sig. |
| 1 | Regression | 191.828 | 4 | 47.957 | 5.388 | <.001^b^ |
|  | Residual | 2144.973 | 241 | 8.900 |  |  |
|  | Total | 2336.801 | 245 |  |  |  |
| a. Dependent Variable: LNS | | | | | | |
| b. Predictors: (Constant), BMI, Sex, Age, APD_dose | | | | | | |

| **Coefficients**^a^ | | | | | | | |
| --- | --- | --- | --- | --- | --- | --- | --- |
| Model | | Unstandardized Coefficients | | Standardized Coefficients | t | Sig. | 95.0% Confidence Interval for B |
|  |  | B | Std. Error | Beta |  |  | Lower Bound |
| 1 | (Constant) | 14.255 | 1.187 |  | 12.010 | <.001 | 11.917 |
|  | APD_dose | -.024 | .013 | -.122 | -1.849 | .066 | -.050 |
|  | Sex | .024 | .392 | .004 | .061 | .952 | -.748 |
|  | Age | .007 | .017 | .026 | .399 | .690 | -.026 |
|  | BMI | -.156 | .045 | -.233 | -3.463 | <.001 | -.245 |

| **Coefficients**^a^ | | | |  |  |  |  |  |
| --- | --- | --- | --- | --- | --- | --- | --- | --- |
| Model | | 95.0% Confidence Interval for B | |  |  |  |  |  |
|  |  | Upper Bound | |  |  |  |  |  |
| 1 | (Constant) | 16.593 | |  |  |  |  |  |
|  | APD_dose | .002 | |  |  |  |  |  |
|  | Sex | .795 | |  |  |  |  |  |
|  | Age | .039 | |  |  |  |  |  |
|  | BMI | -.067 | |  |  |  |  |  |
|  |  |  |  | |  |  |  |  |

| a. Dependent Variable: LNS |
| --- |

**Visual Learning and memory**

| **Descriptive Statistics** | | | | | | |
| --- | --- | --- | --- | --- | --- | --- |
|  | | Statistic | Bootstrap^a^ | | | |
|  |  |  | Bias | Std. Error | 95% Confidence Interval | |
|  |  |  |  |  | Lower | Upper |
| PAL | Mean | 3.86 | .00 | .36 | 3.16 | 4.56 |
|  | Std. Deviation | 5.644 | -.032 | .357 | 4.899 | 6.256 |
|  | N | 268 | 0 | 0 | 268 | 268 |
| APD_dose | Mean | 5.19046 | .05269 | .95586 | 3.52413 | 7.27047 |
|  | Std. Deviation | 15.566878 | -.126906 | 3.589644 | 8.655385 | 22.610101 |
|  | N | 268 | 0 | 0 | 268 | 268 |
| Sex | Mean | .61 | .00 | .03 | .54 | .66 |
|  | Std. Deviation | .489 | -.001 | .007 | .473 | .499 |
|  | N | 268 | 0 | 0 | 268 | 268 |
| Age | Mean | 37.96 | .01 | .76 | 36.44 | 39.48 |
|  | Std. Deviation | 12.115 | -.011 | .391 | 11.353 | 12.849 |
|  | N | 268 | 0 | 0 | 268 | 268 |
| BMI | Mean | 26.04 | .01 | .28 | 25.49 | 26.62 |
|  | Std. Deviation | 4.734 | -.014 | .212 | 4.296 | 5.144 |
|  | N | 268 | 0 | 0 | 268 | 268 |
| a. Unless otherwise noted, bootstrap results are based on 1000 bootstrap samples | | | | | | |

| **Variables Entered/Removed**^a^ | | | |
| --- | --- | --- | --- |
| Model | Variables Entered | Variables Removed | Method |
| 1 | BMI, Sex, Age, APD_dose^b^ | . | Enter |
| a. Dependent Variable: PAL | | | |
| b. All requested variables entered. | | | |

| **Model Summary** | | | | | | | |
| --- | --- | --- | --- | --- | --- | --- | --- |
| Model | R | R Square | Adjusted R Square | Std. Error of the Estimate | Change Statistics | | |
|  |  |  |  |  | R Square Change | F Change | df1 |
| 1 | .443^a^ | .196 | .184 | 5.097 | .196 | 16.071 | 4 |

| **Model Summary** | | | |  |  |  |  |  |
| --- | --- | --- | --- | --- | --- | --- | --- | --- |
| Model | Change Statistics | | |  |  |  |  |  |
|  | df2 | Sig. F Change | |  |  |  |  |  |
| 1 | 263 | <.001 | |  |  |  |  |  |
|  |  |  |  | |  |  |  |  |

| a. Predictors: (Constant), BMI, Sex, Age, APD_dose |
| --- |

| **ANOVA**^a^ | | | | | | |
| --- | --- | --- | --- | --- | --- | --- |
| Model | | Sum of Squares | df | Mean Square | F | Sig. |
| 1 | Regression | 1670.296 | 4 | 417.574 | 16.071 | <.001^b^ |
|  | Residual | 6833.595 | 263 | 25.983 |  |  |
|  | Total | 8503.892 | 267 |  |  |  |
| a. Dependent Variable: PAL | | | | | | |
| b. Predictors: (Constant), BMI, Sex, Age, APD_dose | | | | | | |

| **Coefficients**^a^ | | | | | | | |
| --- | --- | --- | --- | --- | --- | --- | --- |
| Model | | Unstandardized Coefficients | | Standardized Coefficients | t | Sig. | 95.0% Confidence Interval for B |
|  |  | B | Std. Error | Beta |  |  | Lower Bound |
| 1 | (Constant) | -6.787 | 1.935 |  | -3.507 | <.001 | -10.597 |
|  | APD_dose | .039 | .022 | .107 | 1.800 | .073 | -.004 |
|  | Sex | -.317 | .643 | -.027 | -.494 | .622 | -1.583 |
|  | Age | .150 | .027 | .322 | 5.520 | <.001 | .096 |
|  | BMI | .190 | .072 | .159 | 2.644 | .009 | .049 |

| **Coefficients**^a^ | | | |  |  |  |  |  |
| --- | --- | --- | --- | --- | --- | --- | --- | --- |
| Model | | 95.0% Confidence Interval for B | |  |  |  |  |  |
|  |  | Upper Bound | |  |  |  |  |  |
| 1 | (Constant) | -2.977 | |  |  |  |  |  |
|  | APD_dose | .081 | |  |  |  |  |  |
|  | Sex | .949 | |  |  |  |  |  |
|  | Age | .203 | |  |  |  |  |  |
|  | BMI | .331 | |  |  |  |  |  |
|  |  |  |  | |  |  |  |  |

| a. Dependent Variable: PAL |
| --- |

**Social Cognition**

| **Descriptive Statistics** | | | | | | |
| --- | --- | --- | --- | --- | --- | --- |
|  | | Statistic | Bootstrap^a^ | | | |
|  |  |  | Bias | Std. Error | 95% Confidence Interval | |
|  |  |  |  |  | Lower | Upper |
| RME | Mean | 26.02 | -.01 | .29 | 25.46 | 26.57 |
|  | Std. Deviation | 4.880 | -.005 | .213 | 4.454 | 5.288 |
|  | N | 268 | 0 | 0 | 268 | 268 |
| APD_dose | Mean | 5.19046 | .02481 | .93363 | 3.57917 | 7.26517 |
|  | Std. Deviation | 15.566878 | -.364366 | 3.544358 | 8.559742 | 21.964537 |
|  | N | 268 | 0 | 0 | 268 | 268 |
| Sex | Mean | .61 | .00 | .03 | .55 | .67 |
|  | Std. Deviation | .489 | -.001 | .007 | .472 | .498 |
|  | N | 268 | 0 | 0 | 268 | 268 |
| Age | Mean | 37.96 | .04 | .74 | 36.51 | 39.41 |
|  | Std. Deviation | 12.115 | -.021 | .394 | 11.337 | 12.862 |
|  | N | 268 | 0 | 0 | 268 | 268 |
| BMI | Mean | 26.04 | .01 | .29 | 25.48 | 26.65 |
|  | Std. Deviation | 4.734 | -.005 | .224 | 4.281 | 5.163 |
|  | N | 268 | 0 | 0 | 268 | 268 |
| a. Unless otherwise noted, bootstrap results are based on 1000 bootstrap samples | | | | | | |

| **Variables Entered/Removed**^a^ | | | |
| --- | --- | --- | --- |
| Model | Variables Entered | Variables Removed | Method |
| 1 | BMI, Sex, Age, APD_dose^b^ | . | Enter |
| a. Dependent Variable: RME | | | |
| b. All requested variables entered. | | | |

| **Model Summary** | | | | | | | |
| --- | --- | --- | --- | --- | --- | --- | --- |
| Model | R | R Square | Adjusted R Square | Std. Error of the Estimate | Change Statistics | | |
|  |  |  |  |  | R Square Change | F Change | df1 |
| 1 | .418^a^ | .175 | .162 | 4.466 | .175 | 13.924 | 4 |

| **Model Summary** | | | |  |  |  |  |  |
| --- | --- | --- | --- | --- | --- | --- | --- | --- |
| Model | Change Statistics | | |  |  |  |  |  |
|  | df2 | Sig. F Change | |  |  |  |  |  |
| 1 | 263 | <.001 | |  |  |  |  |  |
|  |  |  |  | |  |  |  |  |

| a. Predictors: (Constant), BMI, Sex, Age, APD_dose |
| --- |

| **ANOVA**^a^ | | | | | | |
| --- | --- | --- | --- | --- | --- | --- |
| Model | | Sum of Squares | df | Mean Square | F | Sig. |
| 1 | Regression | 1111.118 | 4 | 277.779 | 13.924 | <.001^b^ |
|  | Residual | 5246.748 | 263 | 19.950 |  |  |
|  | Total | 6357.866 | 267 |  |  |  |
| a. Dependent Variable: RME | | | | | | |
| b. Predictors: (Constant), BMI, Sex, Age, APD_dose | | | | | | |

| **Coefficients**^a^ | | | | | | | |
| --- | --- | --- | --- | --- | --- | --- | --- |
| Model | | Unstandardized Coefficients | | Standardized Coefficients | t | Sig. | 95.0% Confidence Interval for B |
|  |  | B | Std. Error | Beta |  |  | Lower Bound |
| 1 | (Constant) | 35.154 | 1.696 |  | 20.733 | <.001 | 31.816 |
|  | APD_dose | -.058 | .019 | -.184 | -3.058 | .002 | -.095 |
|  | Sex | .014 | .563 | .001 | .025 | .980 | -1.095 |
|  | Age | -.034 | .024 | -.084 | -1.419 | .157 | -.081 |
|  | BMI | -.290 | .063 | -.282 | -4.611 | <.001 | -.414 |

| **Coefficients**^a^ | | | |  |  |  |  |  |
| --- | --- | --- | --- | --- | --- | --- | --- | --- |
| Model | | 95.0% Confidence Interval for B | |  |  |  |  |  |
|  |  | Upper Bound | |  |  |  |  |  |
| 1 | (Constant) | 38.493 | |  |  |  |  |  |
|  | APD_dose | -.021 | |  |  |  |  |  |
|  | Sex | 1.124 | |  |  |  |  |  |
|  | Age | .013 | |  |  |  |  |  |
|  | BMI | -.166 | |  |  |  |  |  |
|  |  |  |  | |  |  |  |  |

| a. Dependent Variable: RME |
| --- |
