# Supplementary material for: Investigating the relationship between Toll-like receptor activity, low-grade inflammation, cognitive deficits, and antipsychotic drug dose in schizophrenia patients: a moderation analysis
Source: Psychol Med. 2026 Mar 3;56:e63. doi: 10.1017/S0033291726103596 (PMC12969209; doi:10.1017/S0033291726103596)
Supplement: Patlola et al. supplementary material [file S0033291726103596sup001.zip › S0033291726103596sup001/Supplementary doc 2 regression TLR2_4_IL8.docx]

**TLR2 activity**

| **Descriptive Statistics** | | | | | | |
| --- | --- | --- | --- | --- | --- | --- |
|  | | Statistic | Bootstrap^a^ | | | |
|  |  |  | Bias | Std. Error | 95% Confidence Interval | |
|  |  |  |  |  | Lower | Upper |
| TLR2_act | Mean | -.0178367 | .0002702 | .0594470 | -.1322547 | .0976147 |
|  | Std. Deviation | .94913044 | -.00359716 | .07067133 | .80419985 | 1.08392465 |
|  | N | 246 | 0 | 0 | 246 | 246 |
| APD_dose | Mean | 5.47989 | -.01072 | .98669 | 3.65512 | 7.57864 |
|  | Std. Deviation | 16.165134 | -.469014 | 3.711158 | 8.758859 | 22.827401 |
|  | N | 246 | 0 | 0 | 246 | 246 |
| Sex | Mean | .61 | .00 | .03 | .56 | .67 |
|  | Std. Deviation | .488 | -.001 | .007 | .469 | .498 |
|  | N | 246 | 0 | 0 | 246 | 246 |
| Age | Mean | 38.05 | -.07 | .75 | 36.47 | 39.42 |
|  | Std. Deviation | 12.096 | -.043 | .395 | 11.280 | 12.811 |
|  | N | 246 | 0 | 0 | 246 | 246 |
| BMI | Mean | 26.02 | .01 | .31 | 25.44 | 26.65 |
|  | Std. Deviation | 4.750 | -.020 | .230 | 4.272 | 5.181 |
|  | N | 246 | 0 | 0 | 246 | 246 |
| a. Unless otherwise noted, bootstrap results are based on 1000 bootstrap samples | | | | | | |

| **Variables Entered/Removed**^a^ | | | |
| --- | --- | --- | --- |
| Model | Variables Entered | Variables Removed | Method |
| 1 | BMI, Sex, Age, APD_dose^b^ | . | Enter |
| a. Dependent Variable: TLR2_act | | | |
| b. All requested variables entered. | | | |

| **Model Summary** | | | | | | | |
| --- | --- | --- | --- | --- | --- | --- | --- |
| Model | R | R Square | Adjusted R Square | Std. Error of the Estimate | Change Statistics | | |
|  |  |  |  |  | R Square Change | F Change | df1 |
| 1 | .178^a^ | .032 | .015 | .94176263 | .032 | 1.962 | 4 |

| **Model Summary** | | | |  |  |  |  |  |
| --- | --- | --- | --- | --- | --- | --- | --- | --- |
| Model | Change Statistics | | |  |  |  |  |  |
|  | df2 | Sig. F Change | |  |  |  |  |  |
| 1 | 241 | .101 | |  |  |  |  |  |
|  |  |  |  | |  |  |  |  |

| a. Predictors: (Constant), BMI, Sex, Age, APD_dose |
| --- |

| **ANOVA**^a^ | | | | | | |
| --- | --- | --- | --- | --- | --- | --- |
| Model | | Sum of Squares | df | Mean Square | F | Sig. |
| 1 | Regression | 6.961 | 4 | 1.740 | 1.962 | .101^b^ |
|  | Residual | 213.747 | 241 | .887 |  |  |
|  | Total | 220.708 | 245 |  |  |  |
| a. Dependent Variable: TLR2_act | | | | | | |
| b. Predictors: (Constant), BMI, Sex, Age, APD_dose | | | | | | |

| **Coefficients**^a^ | | | | | | | |
| --- | --- | --- | --- | --- | --- | --- | --- |
| Model | | Unstandardized Coefficients | | Standardized Coefficients | t | Sig. | 95.0% Confidence Interval for B |
|  |  | B | Std. Error | Beta |  |  | Lower Bound |
| 1 | (Constant) | -.854 | .371 |  | -2.301 | .022 | -1.585 |
|  | APD_dose | .003 | .004 | .044 | .650 | .517 | -.005 |
|  | Sex | .039 | .124 | .020 | .314 | .754 | -.206 |
|  | Age | .007 | .005 | .091 | 1.353 | .177 | -.003 |
|  | BMI | .020 | .014 | .101 | 1.457 | .147 | -.007 |

| **Coefficients**^a^ | | | |  |  |  |  |  |
| --- | --- | --- | --- | --- | --- | --- | --- | --- |
| Model | | 95.0% Confidence Interval for B | |  |  |  |  |  |
|  |  | Upper Bound | |  |  |  |  |  |
| 1 | (Constant) | -.123 | |  |  |  |  |  |
|  | APD_dose | .011 | |  |  |  |  |  |
|  | Sex | .284 | |  |  |  |  |  |
|  | Age | .018 | |  |  |  |  |  |
|  | BMI | .048 | |  |  |  |  |  |
|  |  |  |  | |  |  |  |  |

| a. Dependent Variable: TLR2_act |
| --- |

**TLR4 Regression**

| **Descriptive Statistics** | | | | | | |
| --- | --- | --- | --- | --- | --- | --- |
|  | | Statistic | Bootstrap^a^ | | | |
|  |  |  | Bias | Std. Error | 95% Confidence Interval | |
|  |  |  |  |  | Lower | Upper |
| TLR4_act | Mean | -.0023491 | -.0058585 | .0730791 | -.1436117 | .1393470 |
|  | Std. Deviation | .99101865 | -.01542297 | .12277298 | .74327957 | 1.20874959 |
|  | N | 184 | 0 | 0 | 184 | 184 |
| APD_dose | Mean | 5.89693 | .02835 | 1.28134 | 3.62231 | 8.74894 |
|  | Std. Deviation | 17.227787 | -.645391 | 4.744291 | 7.279044 | 26.095428 |
|  | N | 184 | 0 | 0 | 184 | 184 |
| Sex | Mean | .60 | .00 | .04 | .52 | .67 |
|  | Std. Deviation | .491 | -.001 | .008 | .470 | .501 |
|  | N | 184 | 0 | 0 | 184 | 184 |
| Age | Mean | 37.55 | .06 | .88 | 35.91 | 39.38 |
|  | Std. Deviation | 11.974 | -.030 | .446 | 11.072 | 12.813 |
|  | N | 184 | 0 | 0 | 184 | 184 |
| BMI | Mean | 26.12 | .02 | .36 | 25.44 | 26.83 |
|  | Std. Deviation | 4.990 | -.005 | .248 | 4.472 | 5.437 |
|  | N | 184 | 0 | 0 | 184 | 184 |
| a. Unless otherwise noted, bootstrap results are based on 1000 bootstrap samples | | | | | | |

| **Variables Entered/Removed**^a^ | | | |
| --- | --- | --- | --- |
| Model | Variables Entered | Variables Removed | Method |
| 1 | BMI, Sex, APD_dose, Age^b^ | . | Enter |
| a. Dependent Variable: TLR4_act | | | |
| b. All requested variables entered. | | | |

| **Model Summary** | | | | | | | |
| --- | --- | --- | --- | --- | --- | --- | --- |
| Model | R | R Square | Adjusted R Square | Std. Error of the Estimate | Change Statistics | | |
|  |  |  |  |  | R Square Change | F Change | df1 |
| 1 | .242^a^ | .059 | .038 | .97213265 | .059 | 2.795 | 4 |

| **Model Summary** | | | |  |  |  |  |  |
| --- | --- | --- | --- | --- | --- | --- | --- | --- |
| Model | Change Statistics | | |  |  |  |  |  |
|  | df2 | Sig. F Change | |  |  |  |  |  |
| 1 | 179 | .028 | |  |  |  |  |  |
|  |  |  |  | |  |  |  |  |

| a. Predictors: (Constant), BMI, Sex, APD_dose, Age |
| --- |

| **ANOVA**^a^ | | | | | | |
| --- | --- | --- | --- | --- | --- | --- |
| Model | | Sum of Squares | df | Mean Square | F | Sig. |
| 1 | Regression | 10.565 | 4 | 2.641 | 2.795 | .028^b^ |
|  | Residual | 169.162 | 179 | .945 |  |  |
|  | Total | 179.728 | 183 |  |  |  |
| a. Dependent Variable: TLR4_act | | | | | | |
| b. Predictors: (Constant), BMI, Sex, APD_dose, Age | | | | | | |

| **Coefficients**^a^ | | | | | | | |
| --- | --- | --- | --- | --- | --- | --- | --- |
| Model | | Unstandardized Coefficients | | Standardized Coefficients | t | Sig. | 95.0% Confidence Interval for B |
|  |  | B | Std. Error | Beta |  |  | Lower Bound |
| 1 | (Constant) | -.720 | .419 |  | -1.720 | .087 | -1.547 |
|  | APD_dose | .010 | .004 | .176 | 2.279 | .024 | .001 |
|  | Sex | .038 | .148 | .019 | .259 | .796 | -.253 |
|  | Age | .001 | .006 | .014 | .177 | .860 | -.012 |
|  | BMI | .023 | .016 | .114 | 1.445 | .150 | -.008 |

| **Coefficients**^a^ | | | |  |  |  |  |  |
| --- | --- | --- | --- | --- | --- | --- | --- | --- |
| Model | | 95.0% Confidence Interval for B | |  |  |  |  |  |
|  |  | Upper Bound | |  |  |  |  |  |
| 1 | (Constant) | .106 | |  |  |  |  |  |
|  | APD_dose | .019 | |  |  |  |  |  |
|  | Sex | .329 | |  |  |  |  |  |
|  | Age | .014 | |  |  |  |  |  |
|  | BMI | .054 | |  |  |  |  |  |
|  |  |  |  | |  |  |  |  |

| a. Dependent Variable: TLR4_act |
| --- |

**IL-8 Regression**

| **Descriptive Statistics** | | | | | | |
| --- | --- | --- | --- | --- | --- | --- |
|  | | Statistic | Bootstrap^a^ | | | |
|  |  |  | Bias | Std. Error | 95% Confidence Interval | |
|  |  |  |  |  | Lower | Upper |
| IL8_pla | Mean | 4.37032 | -.00071 | .18815 | 4.02675 | 4.75612 |
|  | Std. Deviation | 2.917268 | -.033916 | .298676 | 2.348344 | 3.505324 |
|  | N | 256 | 0 | 0 | 256 | 256 |
| APD_dose | Mean | 5.35626 | .01935 | .98686 | 3.62732 | 7.53065 |
|  | Std. Deviation | 15.886660 | -.442496 | 3.680724 | 8.575102 | 22.766602 |
|  | N | 256 | 0 | 0 | 256 | 256 |
| Sex | Mean | .61 | .00 | .03 | .55 | .66 |
|  | Std. Deviation | .490 | -.001 | .007 | .473 | .499 |
|  | N | 256 | 0 | 0 | 256 | 256 |
| Age | Mean | 37.85 | .01 | .76 | 36.31 | 39.44 |
|  | Std. Deviation | 12.180 | -.031 | .405 | 11.286 | 12.919 |
|  | N | 256 | 0 | 0 | 256 | 256 |
| BMI | Mean | 26.09 | -.01 | .29 | 25.50 | 26.63 |
|  | Std. Deviation | 4.779 | -.004 | .218 | 4.329 | 5.197 |
|  | N | 256 | 0 | 0 | 256 | 256 |
| a. Unless otherwise noted, bootstrap results are based on 1000 bootstrap samples | | | | | | |

| **Variables Entered/Removed**^a^ | | | |
| --- | --- | --- | --- |
| Model | Variables Entered | Variables Removed | Method |
| 1 | BMI, Sex, Age, APD_dose^b^ | . | Enter |
| a. Dependent Variable: IL8_pla | | | |
| b. All requested variables entered. | | | |

| **Model Summary** | | | | | | | |
| --- | --- | --- | --- | --- | --- | --- | --- |
| Model | R | R Square | Adjusted R Square | Std. Error of the Estimate | Change Statistics | | |
|  |  |  |  |  | R Square Change | F Change | df1 |
| 1 | .152^a^ | .023 | .008 | 2.906240 | .023 | 1.485 | 4 |

| **Model Summary** | | | |  |  |  |  |  |
| --- | --- | --- | --- | --- | --- | --- | --- | --- |
| Model | Change Statistics | | |  |  |  |  |  |
|  | df2 | Sig. F Change | |  |  |  |  |  |
| 1 | 251 | .207 | |  |  |  |  |  |
|  |  |  |  | |  |  |  |  |

| a. Predictors: (Constant), BMI, Sex, Age, APD_dose |
| --- |

| **ANOVA**^a^ | | | | | | |
| --- | --- | --- | --- | --- | --- | --- |
| Model | | Sum of Squares | df | Mean Square | F | Sig. |
| 1 | Regression | 50.162 | 4 | 12.541 | 1.485 | .207^b^ |
|  | Residual | 2120.004 | 251 | 8.446 |  |  |
|  | Total | 2170.166 | 255 |  |  |  |
| a. Dependent Variable: IL8_pla | | | | | | |
| b. Predictors: (Constant), BMI, Sex, Age, APD_dose | | | | | | |

| **Coefficients**^a^ | | | | | | | |
| --- | --- | --- | --- | --- | --- | --- | --- |
| Model | | Unstandardized Coefficients | | Standardized Coefficients | t | Sig. | 95.0% Confidence Interval for B |
|  |  | B | Std. Error | Beta |  |  | Lower Bound |
| 1 | (Constant) | 3.645 | 1.119 |  | 3.257 | .001 | 1.441 |
|  | APD_dose | .020 | .012 | .108 | 1.612 | .108 | -.004 |
|  | Sex | .193 | .376 | .032 | .514 | .608 | -.547 |
|  | Age | .021 | .016 | .088 | 1.337 | .182 | -.010 |
|  | BMI | -.011 | .042 | -.019 | -.274 | .785 | -.093 |

| **Coefficients**^a^ | | | |  |  |  |  |  |
| --- | --- | --- | --- | --- | --- | --- | --- | --- |
| Model | | 95.0% Confidence Interval for B | |  |  |  |  |  |
|  |  | Upper Bound | |  |  |  |  |  |
| 1 | (Constant) | 5.850 | |  |  |  |  |  |
|  | APD_dose | .044 | |  |  |  |  |  |
|  | Sex | .933 | |  |  |  |  |  |
|  | Age | .052 | |  |  |  |  |  |
|  | BMI | .070 | |  |  |  |  |  |
|  |  |  |  | |  |  |  |  |

| a. Dependent Variable: IL8_pla |
| --- |
