# Supplementary material for: Investigating the relationship between Toll-like receptor activity, low-grade inflammation, cognitive deficits, and antipsychotic drug dose in schizophrenia patients: a moderation analysis
Source: Psychol Med. 2026 Mar 3;56:e63. doi: 10.1017/S0033291726103596 (PMC12969209; doi:10.1017/S0033291726103596)
Supplement: Patlola et al. supplementary material [file S0033291726103596sup001.zip › S0033291726103596sup001/Supplementary doc 3 moderation analysis.docx]

**Moderation analysis of TLR4 and APS**

Run MATRIX procedure:

***************** PROCESS Procedure for SPSS Version 4.2 *****************

 Written by Andrew F. Hayes, Ph.D. www.afhayes.com
 Documentation available in Hayes (2022). www.guilford.com/p/hayes3

**************************************************************************
Model : 1
 Y : DSC
 X : TLR4_act
 W : APD_dose

Sample
Size: 189

**************************************************************************
OUTCOME VARIABLE:
 DSC

Model Summary
 R R-sq MSE F df1 df2 p
 .3375 .1139 354.4372 7.9250 3.0000 185.0000 .0001

Model
 coeff se t p LLCI ULCI
constant 72.3297 1.4656 49.3516 .0000 69.4382 75.2211
TLR4_act -2.5359 1.5231 -1.6650 .0976 -5.5408 .4690
APD_dose -.4864 .1089 -4.4651 .0000 -.7013 -.2715
Int_1 .1439 .0619 2.3242 .0212 .0218 .2661

Product terms key:
 Int_1 : TLR4_act x APD_dose

Test(s) of highest order unconditional interaction(s):
 R2-chng F df1 df2 p
X*W .0259 5.4021 1.0000 185.0000 .0212
----------
 Focal predict: TLR4_act (X)
 Mod var: APD_dose (W)

Conditional effects of the focal predictor at values of the moderator(s):

 APD_dose Effect se t p LLCI ULCI
 .0000 -2.5359 1.5231 -1.6650 .0976 -5.5408 .4690
 5.8161 -1.6989 1.4403 -1.1796 .2397 -4.5404 1.1426
 22.8312 .7498 1.6870 .4445 .6572 -2.5784 4.0779

*********************** ANALYSIS NOTES AND ERRORS ************************

Level of confidence for all confidence intervals in output:
 95.0000

W values in conditional tables are the minimum, the mean, and 1 SD above the mean.

NOTE: One SD below the mean is below the minimum observed in the data for W,
 so the minimum measurement on W is used for conditioning instead.

------ END MATRIX -----

**Moderation analysis of TLR4 and SC**

Run MATRIX procedure:

***************** PROCESS Procedure for SPSS Version 4.2 *****************

 Written by Andrew F. Hayes, Ph.D. www.afhayes.com
 Documentation available in Hayes (2022). www.guilford.com/p/hayes3

**************************************************************************
Model : 1
 Y : RME
 X : TLR4_act
 W : APD_dose

Sample
Size: 189

**************************************************************************
OUTCOME VARIABLE:
 RME

Model Summary
 R R-sq MSE F df1 df2 p
 .2888 .0834 22.0735 5.6099 3.0000 185.0000 .0011

Model
 coeff se t p LLCI ULCI
constant 26.4002 .3657 72.1815 .0000 25.6786 27.1218
TLR4_act -.1212 .3801 -.3188 .7502 -.8711 .6287
APD_dose -.0922 .0272 -3.3911 .0009 -.1458 -.0386
Int_1 .0096 .0155 .6240 .5334 -.0208 .0401

Product terms key:
 Int_1 : TLR4_act x APD_dose

Test(s) of highest order unconditional interaction(s):
 R2-chng F df1 df2 p
X*W .0019 .3894 1.0000 185.0000 .5334
----------
 Focal predict: TLR4_act (X)
 Mod var: APD_dose (W)

Conditional effects of the focal predictor at values of the moderator(s):

 APD_dose Effect se t p LLCI ULCI
 .0000 -.1212 .3801 -.3188 .7502 -.8711 .6287
 5.8161 -.0651 .3594 -.1811 .8565 -.7742 .6440
 22.8312 .0990 .4210 .2351 .8144 -.7316 .9295

*********************** ANALYSIS NOTES AND ERRORS ************************

Level of confidence for all confidence intervals in output:
 95.0000

W values in conditional tables are the minimum, the mean, and 1 SD above the mean.

NOTE: One SD below the mean is below the minimum observed in the data for W,
 so the minimum measurement on W is used for conditioning instead.

------ END MATRIX -----

**Multiple T tests between Single APD and Polypharmacy groups**

|  | | Levene's Test for Equality of Variances | | t-test for Equality of Means | | | | |  |
| --- | --- | --- | --- | --- | --- | --- | --- | --- | --- |
|  |  | F | Sig. | t | df | Significance | Mean Difference | Std. Error Difference | |
|  |  |  |  |  |  | Two-Sided p |  |  |  |
| TLR2_act | Equal variances assumed | 1.942 | .168 | 1.203 | 72 | .233 | .30936720 | .25707165 | |
|  | Equal variances not assumed |  |  | 1.346 | 64.679 | .183 | .30936720 | .22991025 | |
| TLR4_act | Equal variances assumed | 3.810 | .056 | .959 | 61 | .342 | .36167737 | .37727449 | |
|  | Equal variances not assumed |  |  | 1.165 | 54.755 | .249 | .36167737 | .31039336 | |
| IL6_pla | Equal variances assumed | 1.265 | .264 | .564 | 74 | .575 | .70378 | 1.24837 | |
|  | Equal variances not assumed |  |  | .758 | 58.009 | .451 | .70378 | .92835 | |
| IL8_pla | Equal variances assumed | .134 | .716 | .322 | 74 | .749 | .291077 | .905060 | |
|  | Equal variances not assumed |  |  | .361 | 64.499 | .719 | .291077 | .805519 | |
| TNF_pla | Equal variances assumed | .795 | .375 | -1.184 | 77 | .240 | -.130789 | .110431 | |
|  | Equal variances not assumed |  |  | -1.080 | 39.749 | .287 | -.130789 | .121062 | |
| IL10_pla | Equal variances assumed | 1.760 | .190 | .607 | 57 | .546 | .811520 | 1.336687 | |
|  | Equal variances not assumed |  |  | .818 | 37.365 | .418 | .811520 | .991518 | |
| IL12_pla | Equal variances assumed | .462 | .499 | .246 | 58 | .806 | .027850 | .113131 | |
|  | Equal variances not assumed |  |  | .287 | 57.622 | .775 | .027850 | .096925 | |
| IFNG_pla | Equal variances assumed | 2.860 | .096 | -1.553 | 62 | .125 | -.213898 | .137727 | |
|  | Equal variances not assumed |  |  | -1.379 | 33.271 | .177 | -.213898 | .155071 | |
| CRP_mgL | Equal variances assumed | 21.717 | <.001 | -2.402 | 72 | .019 | -1.375951 | .572739 | |
|  | Equal variances not assumed |  |  | **-2.049** | **32.873** | **.049** | **-1.375951** | **.671547** | |
| DSC | Equal variances assumed | .430 | .514 | 1.918 | 78 | .059 | 7.439 | 3.879 | |
|  | Equal variances not assumed |  |  | **2.053** | **59.103** | **.044** | **7.439** | **3.623** | |
| WMS | Equal variances assumed | 2.548 | .115 | .327 | 72 | .745 | .932 | 2.853 | |
|  | Equal variances not assumed |  |  | .298 | 34.711 | .768 | .932 | 3.127 | |
| LNS | Equal variances assumed | .618 | .435 | -.648 | 66 | .520 | -.529 | .817 | |
|  | Equal variances not assumed |  |  | -.680 | 43.418 | .500 | -.529 | .778 | |
| FSIQ | Equal variances assumed | 1.603 | .209 | 1.754 | 79 | .083 | 6.821 | 3.888 | |
|  | Equal variances not assumed |  |  | 1.941 | 63.684 | .057 | 6.821 | 3.515 | |
| RME | Equal variances assumed | .551 | .460 | 1.806 | 79 | .075 | 2.100 | 1.163 | |
|  | Equal variances not assumed |  |  | 1.907 | 56.523 | .062 | 2.100 | 1.101 | |
| PAL | Equal variances assumed | 1.175 | .282 | -.818 | 79 | .416 | -1.508 | 1.844 | |
|  | Equal variances not assumed |  |  | -.794 | 45.609 | .432 | -1.508 | 1.901 | |
| HAM_D | Equal variances assumed | .234 | .630 | -2.159 | 79 | .034 | -2.187 | 1.013 | |
|  | Equal variances not assumed |  |  | **-2.033** | **42.497** | **.048** | **-2.187** | **1.076** | |
| DOI | Equal variances assumed | 7.021 | .010 | -2.708 | 76 | .008 | -6.605 | 2.439 | |
|  | Equal variances not assumed |  |  | -3.085 | 65.536 | .003 | -6.605 | 2.141 | |
| Sex | Equal variances assumed | .401 | .528 | .330 | 79 | .743 | .037 | .112 | |
|  | Equal variances not assumed |  |  | .325 | 47.422 | .747 | .037 | .114 | |
| Age | Equal variances assumed | .729 | .396 | -2.026 | 79 | .046 | -5.131 | 2.532 | |
|  | Equal variances not assumed |  |  | -2.104 | 54.123 | .040 | -5.131 | 2.438 | |
| BMI | Equal variances assumed | 2.556 | .114 | -2.559 | 77 | .012 | -2.936 | 1.147 | |
|  | Equal variances not assumed |  |  | **-2.373** | **41.288** | **.022** | **-2.936** | **1.237** | |
