# Supplementary material for: Investigating the relationship between Toll-like receptor activity, low-grade inflammation, cognitive deficits, and antipsychotic drug dose in schizophrenia patients: a moderation analysis
Source: Psychol Med. 2026 Mar 3;56:e63. doi: 10.1017/S0033291726103596 (PMC12969209; doi:10.1017/S0033291726103596)
Supplement: Patlola et al. supplementary material [file S0033291726103596sup001.zip › S0033291726103596sup001/Supplementary methods APD.docx]

**1. Demographic data**

*1.1. Exclusion criteria for patients:*

(1) A chronic inflammatory illness

(2) Taken non-steroidal anti-inflammatory drugs (NSAIDs) in the past 24 hrs

(3) a history of acquired brain injury causing loss of consciousness of more than one minute (4) substance abuse in the preceding six months of the study

(5) IQ < 70

(6) Diagnosis of a neurological disorder (e.g. epilepsy).

*1.1. Exclusion criteria for controls:*

(1) No history of psychiatric or neurodegenerative disorders

(2) No general health problems

(3) No first-degree relatives with schizophrenia spectrum disorders.

**2. ELISA**

*2.1. Quantikine-high sensitive ELISA (Bio-Techne Ltd.; R&D Systems)*

*2.1.1. IL-6, TNF-α & IFN-γ*

The reagents were prepared according to the company provided protocol. Firstly, wash buffer concentrate; (PART# 895003) was warmed to room temperature (RT) and mixed gently to dissolve the crystals. 40 millilitres (mL) of this concentrate was added to 960 mL of distilled water to prepare 1000 mL of wash buffer. Streptavidin solution was prepared by 100-fold dilution of the streptavidin concentrate (Streptavidin Polymer-HRP (100X); PART# 898350) in the diluent (Streptavidin Polymer-HRP Diluent; PART# 898387). Human cytokine HS standard (IL-6: PART# 898935, TNF-α: PART# 898532, IFN-γ: PART# 899181) was used to prepare series of standards. Stock standards were diluted in calibrator diluent (IL-6: RD5-4, PART# 895435; TNF-α: RD6S, PART# 895142; IFN-γ: RD5P, PART# 895151) in a 1.5 mL centrifuge tube and a serial dilution was performed to make seven point standards and a blank (0 pg/mL). A pre-coated microplate-96well containing human cytokine specific monoclonal antibody (IL-6: PART# 898933; TNF-α: PART# 898530, IFN-γ: PART# 899179) was used. Initially, 100 uL (for IL-6 & IFN-γ) or 50 uL (for TNF-α) of assay diluent (IL-6: RD1W, PART# 895117; TNF-α: RD1-40, PART# 895513; IFN-γ: RD1-63, PART# 895352) was added to each well followed by 100 µL (for IL-6 & IFN-γ) or 50 µL (for TNF-α) of standards and samples per well in duplicates which was sealed by adhesive strip/plate cover. The plate was incubated on a microplate shaker at 500 rpm for 2 hours at RT. After incubation, the contents in the well were aspirated by a plate washer and washed by filling the well with 400 uL of wash buffer. This wash step was repeated for 3 more times and then the plate was inverted to blot on a clean paper and any remaining liquid was removed. 200 uL of human cytokine HS conjugate (IL-6: PART# 898934; TNF-α: PART# 898530, IFN-γ: PART# 899180) was added to each well, covered with a new plate cover and incubated for 1 hour at RT on the plate shaker. This was followed by the wash step and 200 µL of streptavidin was added to each well, covered with a new plate cover and incubated for 1 hour at RT on the plate shaker. Wash step was repeated and 200 µL of substrate solution which was made by mixing colour reagents A & B (PART# 895000 & 895001) was added to each well, covered with a tin/aluminium foil to protect from light and incubated for 30 minutes at RT for the development. 50 µL of stop solution (2N sulphuric acid; PART# 895032) was added to each well where the colour changes from blue to yellow. Gentle taps to the plate to ensure thorough mixing was carried out. The plate was read at 450 nm under the plate reader and the absorbance values were recorded and exported in to an excel spreadsheet. The sensitivity of this assay was 0.007- 0.09 pg/mL (IL-6), 0.011- 0.049 pg/mL (TNF-α) and 0.025- 0.173 pg/mL (IFN-γ).

**SM Table 1: S**erial dilutions and concentrations for standards for the Quantikine-high sensitive ELISA assays.

| Cytokine | Volume of stock standard (µL) | Concentration of HS standard (pg/mL) | Volume of diluent (µL) | Serial dilution concentrations of standards (pg/mL) |
| --- | --- | --- | --- | --- |
| IL-6 | 100 | 100 | 900 | 10, 5, 2.5, 1.25, 0.625, 0.313, 0.156, 0 |
| TNF-α | 50 | 100 | 450 | 10, 5, 2.5, 1.25, 0.625, 0.313, 0.156, 0 |
| IFN-γ | 100 | 300 | 900 | 30, 15, 7.5, 3.75, 1.88, 0.94, 0.47, 0 |
| IL-8 | 100 | 640 | 900 | 64, 32, 16, 8, 4, 2, 1, 0 |
| IL-10 | 100 | 500 | 900 | 50, 25, 12.5, 6.25, 3.13, 1.56, 0.78, 0 |

*2.1.2. IL-8 & IL-10*

The reagents were prepared according to the company provided protocol. Firstly, wash buffer concentrate; (PART# 895188) was warmed to room temperature (RT) and mixed gently to dissolve the crystals. 100 millilitres (mL) of this concentrate was added to 900 mL of distilled water to prepare 1000 mL of wash buffer. Amplifier solution was prepared by reconstituting the lyophilised amplifier (PART# 895886) in the diluent (amplifier Diluent; PART# 895887). Human cytokine HS standard (IL-8: PART# 894084; IL-10: PART# 893859) was used to prepare series of standards. 100 microliters (µL) of 640 and 500 pg/mL stock standard for IL-8 and IL-10 respectively was transferred to 900 µL of calibrator diluent (IL-8: RD6Z, PART# 895466; IL-10: RD6-10; PART# 895468) in a 1.5 mL centrifuge tube to make first standard. A serial dilution was performed to prepare 7 point standards and a blank (0 pg/mL). A pre-coated microplate-96well containing human cytokine specific monoclonal antibody (IL-8: PART# 894082; IL-10: PART# 893857) was used. Initially, 100 µL of assay diluent (IL-8: RD1-85; PART# 895877; IL-10: RD1-10; PART# 895168) was added to each well followed by 100 µL of standards and samples per well in duplicates which was sealed by adhesive strip/plate cover. The plate was incubated on a microplate shaker at 500 rpm for 2 hours at RT. After incubation, the contents in the well were aspirated by a plate washer and washed by filling the well with 400 µL of wash buffer. This wash step was repeated for 5 more times and then the plate was inverted to blot on a clean paper and any remaining liquid was removed. 200 µL of human cytokine HS conjugate (IL-8: PART# 894083; IL-10: PART# 893858) was added to each well, covered with a new plate cover and incubated for 1 hour at RT on the plate shaker. This was followed by the wash step and 50 µL of substrate made by diluting substrate concentrate (PART# 895884) with substrate dilute (PART# 895885) which was added to each well, covered with a new plate cover and incubated for 1 hour at RT, covered with a tin/aluminium foil to protect from light and incubated. 50 µL amplifier solution was added to each well. 50 µL of stop solution (2N sulphuric acid; PART# 895032) was added to each well Gentle taps to the plate to ensure thorough mixing was carried out. The plate was read at 490 nm under the plate reader and the absorbance values were recorded and exported in to an excel spreadsheet. The sensitivity of this assay was 0.02- 0.4 pg/mL (IL-8) and 0.03- 0.17 pg/mL (IL-10).

*2.2. Quantikine ELISA (Bio-Techne Ltd.; R&D Systems)*

*2.2.1. C-Reactive protein*

The reagents were prepared according to the company provided protocol. Firstly, wash buffer concentrate; (PART# 895003) was warmed to room temperature (RT) and mixed gently to dissolve the crystals. 40 millilitres (mL) of this concentrate was added to 960 mL of distilled water to prepare 1000 mL of wash buffer. Calibrator diluent (RD5P) was prepared by adding 20 mL of RD5P concentrate to 80 mL distilled water. Human CRP standard (PART# 893169) was used to prepare series of standards. Stock standard (50 ng/mL) were diluted in calibrator diluent (RD5P) in a 1.5 mL centrifuge tube and a serial dilution was performed to make seven point standards (25, 12.5, 6.25, 3.13, 1.56, 0.78 ng/mL) and a blank (0 ng/mL). A pre-coated microplate-96well containing human CRP specific monoclonal antibody (PART# 893167) was used. Initially, 100 µL of assay diluent (RD1F, PART# 895041) was added to each well followed by 50 µL of standards and samples per well in duplicates which was sealed by adhesive strip/plate cover. The plate was incubated for 2 hours at RT. After incubation, the contents in the well were aspirated by a plate washer and washed by filling the well with 400 uL of wash buffer. This wash step was repeated for 3 more times and then the plate was inverted to blot on a clean paper and any remaining liquid was removed. 200 µL of human CRP conjugate (PART# 898168) was added to each well, covered with a new plate cover and incubated for 2 hour at RT. This was followed by the wash step and 200 µL of substrate solution which was made by mixing colour reagents A & B (PART# 895000 & 895001) was added to each well, covered with a tin/aluminium foil to protect from light and incubated for 30 minutes at RT for the development. 50 µL of stop solution (2N sulphuric acid; PART# 895032) was added to each well where the colour changes from blue to yellow. Gentle taps to the plate to ensure thorough mixing was carried out. The plate was read at 450 nm under the plate reader and the absorbance values were recorded and exported in to an excel spreadsheet. The sensitivity of this assay was 0.005- 0.022 ng/mL.

*2.3. Duoset ELISA (Bio-Techne Ltd.; R&D Systems)*

*2.3.1. Whole blood stimulated cytokines quantification*

DuoSet ELISA kits provided by the company (Bio-Techne Ltd.; R&D Systems) were utilised to quantify the cytokines: IL-6 (Catalog# DY206), IL-8 (Catalog# DY208), IL-10 (Catalog# DY217B) and TNF-α (Catalog# DY210) in the stimulated blood samples. The reagents were prepared according to the company protocol and a pH of 7.2-7.4 was maintained for all the reagents/solutions. Phosphate buffer saline (PBS) solution was made by adding 1 PBS tablet (Sigma Aldrich, Catalog# P4417) for every 200 mL of distilled water. Wash buffer (with 0.05% Tween 20) was made by adding 0.5 µL of Tween 20 (Sigma Aldrich, Catalog# P1379) to every 1 L of PBS. Reagent diluent (1% BSA) was made by adding 1 g of heat shock fraction of bovine serum albumin (Sigma Aldrich, Catalog# A8022) to every 100 mL of PBS. Capture antibody, detection antibody, cytokine standards and streptavidin were reconstituted according to the manual. A seven point standards were made using the reconstituted stock standards and diluting them in reagent diluent. The concentrations ranged from 600 to 9.38 pg/mL for IL-6, 2000 to 31.3 pg/mL for IL-8 & IL-10 and 1000 to 15.6 pg/mL for TNF-α. Before starting ELISA all the reagents were brought to room temperature.

Capture antibody was diluted in PBS to the working concentration and 100 µL of the same was added to each well on a 96 well plate (Maxisorp Nunc-immuno 96well plates; Thermo Fisher scientific, Catalog# 439454), sealed with plate cover and left overnight at room temperature (RT). The contents were aspirated using an automatic plate washer while simultaneously wash buffer was dispensed into the wells. This procedure was repeated for a total of 3 times and the plate was blotted against a clean paper towel to remove any remaining wash buffer. The plate was then blocked using 300 µL of reagent diluent for a minimum of 1 hour at RT and followed by wash step. To the plate, 100 µL of standards and samples were added in duplicates, sealed and incubated for 2 hours at RT. The wash step was repeated and detection antibody was diluted to working concentration and 100 µL of the same was added to each well, sealed and incubated for 2 hours at RT. The wash step was repeated and 100 µL of the streptavidin (working concentration) was added to each well, covered with tin/aluminium foil to prevent direct light and incubated for 20 minutes at RT. The wash step was repeated and 100 µL of the substrate (3,3',5,5'-Tetramethylbenzidine, supersensitive; Sigma Aldrich, Catalog# T4444) was added to each well, covered with tin/aluminium foil and incubated for 20 more minutes at RT. To this, 50 µL of stop solution (2N sulphuric acid) was added to each well and plate was gently tapped to ensure mixing of the contents. The plate was then read at 450 nm under a plate reader and the absorbance values were recorded and exported into an excel spreadsheet.

**3. Cognition Tasks**

*3.1. Full scale intelligence quotient (FSIQ)*

FSIQ is a measure of combined scores from performance intelligence and verbal intelligence.

*3.2. Digit Symbol Coding (DSC)*

This task is used to assess the processing speed and execution ability of the participant. In this task, a reference panel consisting of numbers 0-9 and their paired symbols. The participant is given 2 practice tests where they have to recode 5 numbers into symbols and vice versa respectively. Then the timed assessment starts where the participant has to recode a series of randomly arranged numbers and symbols within 120 seconds. They are scored 0-133.

*3.3. Logical memory (LM)*

This task is a combination of 2 subtasks namely, LM 1 (immediate recall) and LM2 (delayed recall) assessing short term and long term memories respectively. In this study only the former was administrated. This task (LM1) involves narration of two stories and the participants are asked to recall them immediately. They are scored from 0-75.

*3.4. Paired Associates Learning (PAL)*

This task is used to assess each participant’s visual memory and ability to learn new things. In this task, 6 boxes are displayed that open in random order and one or more boxes contain a pattern. Later, patterns are displayed at the centre and the participant has to choose the right box. The number of errors and attempts are recorded. Total errors 6 Shapes (Adjusted) scores were used for the assessment.

*3.5. Letter-Number Sequencing (LNS)*

This task is used to assess working memory capacity. This task involves participants to listen to a combination of letters and numbers (item) and then repeat back the numbers first in ascending order and then letters in alphabetical order. Each item is scored 1 and repeated thrice with different characters. The string length for the 1^st^ item is 2 characters and then they increase by 1 each time upto a final of 8 characters. In total 21 items are delivered for a maximum score of 21.

*3.6. Reading the Mind in the Eyes (RME)*

This task measures the ability to identify emotional expressions from the eyes of others which is “Theory of mind”. The participants are shown 36 items (photographs) of human eyes and are given 4 emotional expressions (1 correct answer) and are asked to choose one for each item. The scores range from 0 – 36.
