# Supplementary material for: Investigating the relationship between Toll-like receptor activity, low-grade inflammation, cognitive deficits, and antipsychotic drug dose in schizophrenia patients: a moderation analysis
Source: Psychol Med. 2026 Mar 3;56:e63. doi: 10.1017/S0033291726103596 (PMC12969209; doi:10.1017/S0033291726103596)
Supplement: Patlola et al. supplementary material [file S0033291726103596sup001.zip › S0033291726103596sup001/Supplementary tables.docx]

**Supplementary Table 1: Domains of cognition and tasks included in them**

| **Cognition Domain** | **Task** |
| --- | --- |
| Full-scale intelligence quotient (FSIQ) | Wechsler Adult Intelligent Scale, 3rd edition |
| Attention & processing speed (APS) | Digit symbol coding |
| Verbal learning and memory (VLM) | Logical memory subset |
| Visual learning and memory (VisLM) | Paired associates learning (error scores) |
| Working memory (WM) | Letter number sequencing |
| Social cognition (SC) | Reading of the mind in the eyes test |

**Supplementary Table 2. Breakdown of antipsychotic drug intake between participants.**

| **Category** | **Participants** |
| --- | --- |
| 1 APD | 54 |
| 2 APDs | 22 |
| 3 APDs | 3 |
| Clozapine | 32 |
| Olanzapine | 18 |
| Aripiprazole | 17 |
| Quetiapine | 11 |
| Paliperidone | 11 |
| Risperidone | 10 |
| Other APDs, including 1^st^ generation | 12 |

APDs – Antipsychotic drugs
